# Three-Dimensional Printed Materials for Definitive Ceramic and Resin Restorations: A Scoping Review

**DOI:** 10.3390/ma19173728

**Published:** 2026-09-01

**Authors:** Ioulianos Rachiotis, Aspasia Pachiou, Maurice Salem, Duygu Karasan, Irena Sailer, Christos Rahiotis

**Affiliations:** 1 Dental School, National and Kapodistrian University of Athens, Thivon 2, 11527 Athens, Greece; sdn2100077@uoa.gr; 2 Clinic of Reconstructive Dentistry, Center for Dental Medicine, University of Zurich, Plattenstrasse 11, 8032 Zurich, Switzerland; aspasia.pachiou@zzm.uzh.ch; 3 Division of Fixed Prosthodontics and Biomaterials, University Clinics for Dental Medicine, University of Geneva, 1211 Geneva, Switzerland; maurice.salem@unige.ch (M.S.); duygu.narin-karasan@unige.ch (D.K.); irena.sailer@unige.ch (I.S.); 4 Department of Operative Dentistry, Dental School, National and Kapodistrian University of Athens, Thivon 2, 11527 Athens, Greece

**Keywords:** three-dimensional printing, additive manufacturing, dental materials, digital dentistry

## Abstract

Background: The integration of additive manufacturing (AM) into digital dentistry has extended 3D printing beyond auxiliary applications toward the fabrication of definitive restorations, yet the evidence remains dispersed across diverse materials and technologies. This scoping review aimed to map the available evidence on 3D-printed resin- and ceramic-based materials for definitive tooth-supported restorations, including crowns, fixed dental prostheses, endocrowns, veneers, overlays, and onlays. Methods: The review followed the JBI framework and reported according to PRISMA-ScR. MEDLINE, Embase, and Scopus were searched from inception to April 2026. Two reviewers screened records independently against predefined eligibility criteria, and data were extracted using a piloted charting form capturing study design, materials, additive manufacturing technology, restoration type, and evaluated outcomes. Given the anticipated heterogeneity, extracted data were synthesised descriptively and mapped narratively rather than pooled statistically; 111 studies met the inclusion criteria. Results: The evidence was predominantly in vitro (105 studies), with only six clinical studies, including one randomised controlled trial. Research concentrated on single crowns, zirconia and ceramic-filled/methacrylate-based resins, and vat-photopolymerization technologies. Additively manufactured restorations were frequently reported as comparable to milled controls in dimensional accuracy, marginal and internal fit, and fracture resistance, with values generally falling within the clinically accepted thresholds defined by the primary studies; reported outcomes were most consistent for printed zirconia, whereas printed resins were more often limited by lower strength and colour instability. Outcomes were strongly influenced by processing parameters, underscoring the need for workflow standardisation. Conclusions: The current evidence supports the technical feasibility of 3D-printed definitive restorations more strongly than their long-term clinical durability, highlighting a need for long-term clinical trials.

## 1. Introduction

The widespread integration of digital workflows in modern dentistry has driven substantial interest in computer-aided design and computer-aided manufacturing (CAD/CAM) technologies, which traditionally rely on either subtractive or additive methodologies [1,2]. Subtractive manufacturing, specifically numerical control (NC) milling, has long served as the established gold standard for fabricating a wide array of definitive dental prostheses [3]. Within this subtractive workflow, yttria-stabilised tetragonal polycrystal zirconia (Y-TZP) and lithium disilicate have become the predominant ceramic frameworks due to their exceptional mechanical properties, biocompatibility, and superior optical aesthetics when compared to traditional metal-ceramic options [4,5,6,7,8]. Additionally, digital subtractive technologies have facilitated the use of resin-matrix ceramics as viable alternatives for fabricating single-piece endodontic crowns [9].

Despite its widespread application and ability to produce high-strength materials with low surface roughness, subtractive milling has several inherent limitations [10]. The milling process is frequently associated with significant material wastage and demands the continuous replacement of specialised milling burs, making the procedure inefficient and heavily reliant on operator expertise [11]. Furthermore, subtractive techniques struggle with the creation of geometrically complex architectures, such as severe undercuts or ultra-thin laminate veneers, and are susceptible to introducing microscopic cracks during the fabrication phase [10,12,13].

To overcome these manufacturing challenges, additive manufacturing (AM), commonly known as three-dimensional (3D) printing, has rapidly gained traction among dental professionals as an innovative and highly flexible alternative [10,14,15,16]. This technology builds objects layer-by-layer, allowing for unparalleled personalised customisation and significantly enhancing both the efficiency and accuracy of definitive dental prostheses [17,18]. Consequently, 3D printing has gained prominence for its precision, design flexibility, faster production speeds, and substantial reductions in material waste and environmental pollution compared to traditional milling [19,20,21,22].

Historically and in current standard prosthetic practice, the use of additively manufactured polymers has been predominantly limited to auxiliary purposes, including the fabrication of working dental casts, custom trays, and surgical guides [23,24,25]. Additionally, 3D-printed resin materials have proven highly effective for creating interim fixed crowns and partial dentures, demonstrating promising accuracy and fabrication trueness [26,27].

While traditionally limited to the above use, as additive manufacturing continues to advance, the fabrication of 3D-printed ceramic and resin definitive restorations has gathered widespread attention, offering the potential to construct highly intricate structures that bypass the limitations of milling [22]. Stereolithographic methods, including stereolithography (SLA) and digital light processing (DLP), have demonstrated strong potential for generating zirconia-based prostheses while conserving material [28,29,30]. This additive approach to ceramics enables the successful fabrication of complex geometries, including ultra-thin laminate veneers that would be otherwise difficult to produce with subtractive machinery [31,32]. Alongside ceramics, light-curing composite resins are increasingly being utilised as 3D-printable restorative materials [33,34]. Compared with printed ceramics, printed composite resins have a lower elastic modulus and hardness, which favourably reduces wear on opposing natural dentition while simultaneously offering easier fabrication, improved marginal adaptation, and superior reparability in the event of a minor fracture [33,34]. However, the ultimate clinical success of these restorations relies heavily on the reliability and accuracy of the production process, as the layer-by-layer polymerisation inherent in 3D printing can influence interlayer bonding, crack propagation patterns, and overall fracture resistance [34,35,36,37,38].

Existing reviews in this field have generally addressed a single material class or a narrow set of outcomes: for example, prior work has focused specifically on additively manufactured composite resins [13] or on printed zirconia in isolation [22]. To date, no review has mapped both printable ceramic and resin materials across the full range of definitive tooth-supported restoration types—including crowns, fixed dental prostheses, endocrowns, veneers, overlays, and inlays/onlays. This cross-material, cross-restoration-shape perspective is needed to identify where research attention has concentrated and where it remains sparse, and, as this review’s findings ultimately show, to reveal a widening gap between laboratory-demonstrated feasibility and the still-limited clinical evidence base. Accordingly, the primary objective of this scoping review is to systematically map and synthesise the evidence regarding 3D-printed resin- and ceramic-based materials intended for definitive tooth-supported fixed dental prostheses, spanning both material families and the full spectrum of restoration types.

## 2. Materials and Methods

### 2.1. Protocol and Reporting Guidelines

This scoping review followed the general reporting principles of the PRISMA 2020 statement [39], was conducted in accordance with the Joanna Briggs Institute (JBI) methodology for scoping reviews [40], and is reported according to the PRISMA extension for Scoping Reviews (PRISMA-ScR) [41]. The review protocol was developed in March 2026, and the literature search was conducted on 4 April 2026. Title and abstract screening and full-text screening were performed between 5 April and 23 July 2026. The protocol was registered with the Open Science Framework (OSF; Registration DOI: 10.17605/OSF.IO/NPC7A) on 24 July 2026, after the search and screening had been completed but before data charting and synthesis began. Accordingly, this review should be considered retrospectively registered with respect to the search and screening stages; however, the eligibility criteria and search strategy had been finalised in the protocol prior to the search and were not modified thereafter, and the data-charting and synthesis stages were conducted only after, and in accordance with, the registered protocol.

### 2.2. Eligibility Criteria

The eligibility criteria were established according to the Population–Concept–Context (PCC) framework recommended for scoping reviews.

The Population comprised teeth and dental substrates—evaluated either in laboratory (in vitro) settings or in patients—receiving a definitive, tooth-supported, additively manufactured restoration. The Context was unrestricted with respect to setting or geographic location; only English-language publications were eligible.

The concept of interest comprised three-dimensional (3D)-printed resin- and ceramic-based materials intended for the fabrication of definitive tooth-supported restorations. Eligible restorations included single crowns, fixed dental prostheses (FDPs), endocrowns, veneers, occlusal table-tops, overlays, and onlays. Studies evaluating additively manufactured restorations fabricated using any printing technology, including stereolithography (SLA), digital light processing (DLP), liquid-crystal display (LCD), material jetting, fused deposition modelling (FDM), binder jetting, selective laser sintering (SLS), lithography-based ceramic manufacturing (LCM), or other additive manufacturing technologies, were considered eligible.

Experimental laboratory studies, animal studies, clinical studies, case series, and case reports evaluating the performance of definitive 3D-printed resin- or ceramic-based tooth-supported restorations, as defined above, were included.

Studies were excluded if they:

- investigated temporary restorations;
- evaluated standardised material specimens only (e.g., discs, bars, blocks) without fabrication of a definitive restoration;
- primarily assessed adhesive or bond strength, cementation protocols, repair procedures, repair materials, or repair bond strength;
- investigated implant-supported restorations;
- evaluated metallic additive manufacturing materials (e.g., cobalt–chromium or titanium);
- investigated removable prostheses, surgical guides, orthodontic appliances, dental models, or maxillofacial prostheses;
- were reviews, conference abstracts, editorials, letters, or opinion articles.

No restrictions were applied regarding publication year. Only studies published in English were considered.

### 2.3. Information Sources and Search Strategy

A comprehensive electronic literature search was performed in MEDLINE (via PubMed), Embase, and Scopus from database inception (until 4 April 2026). The search strategy combined controlled vocabulary and free-text terms related to additive manufacturing, three-dimensional printing, printable resin and ceramic restorative materials, crowns, and fixed dental prostheses. The review team developed the search strategy and did not formally peer-review it before execution. The complete search strategy for each database is provided in the Appendix A.

Additionally, the reference lists of all included studies and relevant reviews were manually screened independently by two reviewers (I.R. and M.S.) to identify potentially eligible publications not retrieved through the electronic search.

### 2.4. Study Selection

**Figure 1:**
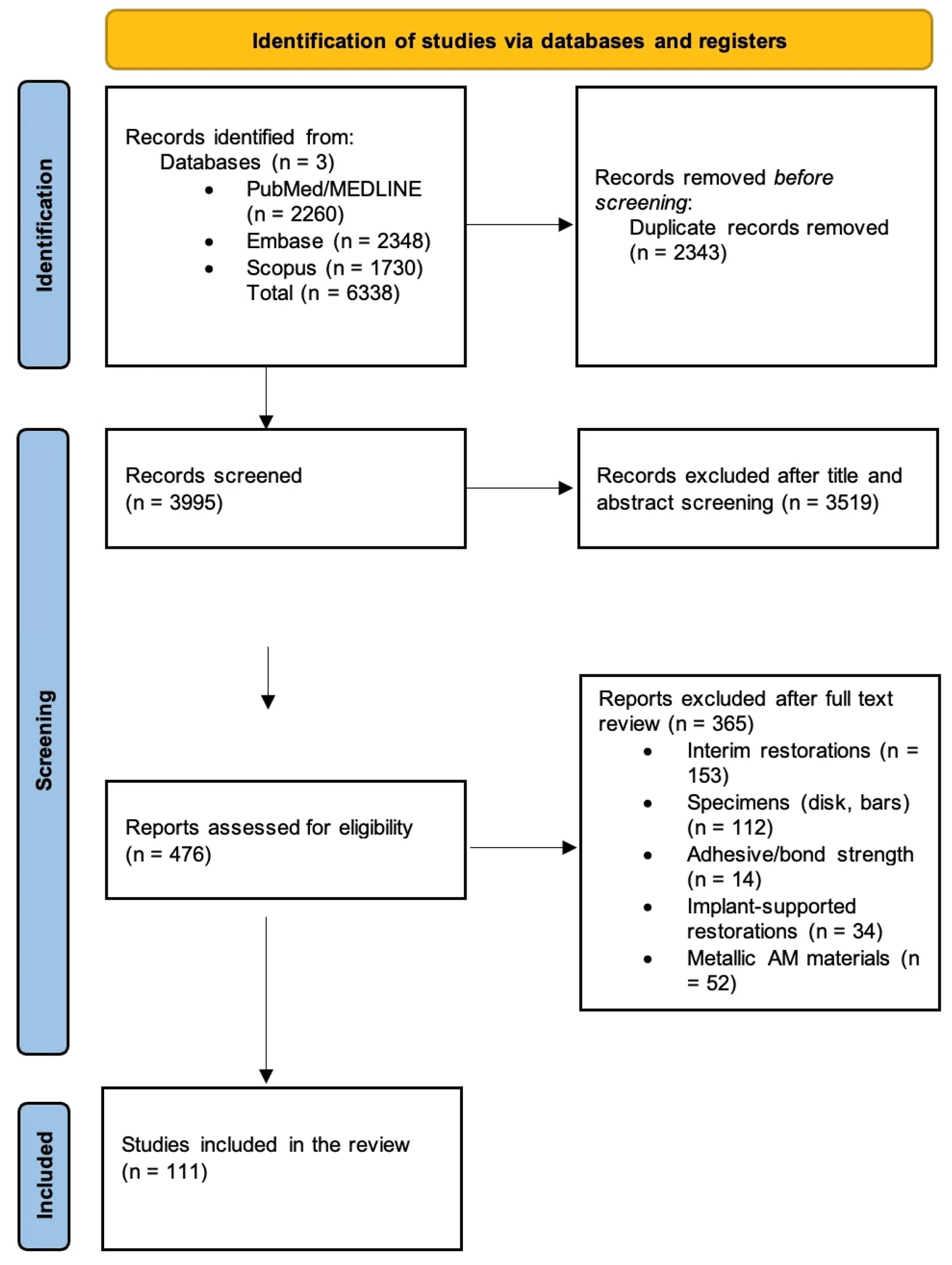
PRISMA 2020 flow diagram.

Duplicate records were identified and removed, and title and abstract screening was managed using Rayyan (Qatar Computing Research Institute). Two independent reviewers (I.R. and M.S.) then screened titles and abstracts for potential eligibility. Full texts of potentially relevant articles were then assessed independently against the predefined inclusion and exclusion criteria. Standardisation across reviewers was ensured by applying predefined, explicit inclusion and exclusion criteria independently. Disagreements were resolved through discussion and, when necessary, consultation with a third reviewer (A.P.). The study selection process is presented using a PRISMA flow diagram (Figure 1).

### 2.5. Data Extraction

Two reviewers (I.R. and A.P.) performed data extraction independently and in duplicate using a common data-charting form. The form was piloted informally by the two reviewers on a subset of included studies and refined by consensus before full-scale extraction to improve clarity and consistency. Reviewers resolved discrepancies through discussion and, when necessary, consultation with a third reviewer. Where a field was not reported in the primary study, this was recorded explicitly as “not reported” (NR) in the charting form. The following information was collected:

- bibliographic characteristics (author, year, country);
- study design;
- restoration type (single crown or FDP);
- printable material evaluated;
- additive manufacturing technology and associated process parameters (e.g., printer/manufacturer, build orientation, and layer thickness);
- post-processing procedures;
- comparator material or manufacturing technique;
- evaluated outcomes and their operational definitions, as reported by the primary study;
- principal findings.

Where available, additional information was extracted regarding fracture resistance, fatigue behaviour, marginal and internal adaptation, dimensional accuracy, surface roughness, wear resistance, optical properties, ageing performance, cytocompatibility, antimicrobial properties, and clinical outcomes. Consistent with the descriptive, mapping-oriented objective of this scoping review, sample size (at the specimen, patient, or restoration level), funding source, follow-up duration, and numerical outcome values with a corresponding measure of uncertainty (e.g., mean ± standard deviation or 95% confidence interval) were not systematically charted as discrete fields. Study authors were not contacted to request missing or unclear data.

### 2.6. Data Synthesis

As the objective of this scoping review was to map the current evidence regarding 3D-printed resin- and ceramic-based materials for definitive tooth-supported crowns and fixed dental prostheses, no formal risk-of-bias assessment or quantitative meta-analysis was performed. A quantitative synthesis was considered inappropriate because of substantial heterogeneity in study designs, evaluated outcomes, testing methodologies, and reporting approaches across the included studies. Extracted data were synthesised descriptively using tables and narrative summaries. Studies were categorised according to printable material, additive manufacturing technology, study design, restoration type, and evaluated outcomes to identify current research trends, evidence gaps, and priorities for future research.

## 3. Results

### 3.1. Study Selection

An electronic search of MEDLINE (via PubMed), Embase, and Scopus, complemented by manual screening of reference lists, retrieved 6338 studies. Following the removal of 2343 duplicates, 3995 titles and abstracts were screened, of which 3519 were excluded. A total of 476 full-text articles were assessed for eligibility, and 365 were excluded for the following reasons: temporary restorations (n = 153); specimen-only evaluations (n = 112); adhesive/bond-strength (n = 14); implant-supported restorations (n = 34); metallic AM materials (n = 52). Ultimately, 111 studies met the inclusion criteria and were incorporated into the qualitative synthesis. The study selection process is summarised in the PRISMA flow diagram (Figure 1). Full study characteristics and extracted data for each included study are provided in Supplementary Table S1 (ceramic-based restorations), Supplementary Table S2 (resin-based restorations), and Supplementary Table S3 (studies directly comparing additively manufactured zirconia and resin materials) [20,42,43,44,45,46,47,48,49,50,51,52,53,54,55,56,57,58,59,60,61,62,63,64,65,66,67,68,69,70,71,72,73,74,75,76,77,78,79,80,81,82,83,84,85,86,87,88,89,90,91,92,93,94,95,96,97,98,99,100,101,102,103,104,105,106,107,108,109,110,111,112,113,114,115,116,117,118,119,120,121,122,123,124,125,126,127,128,129,130,131,132,133,134,135,136,137,138,139,140,141,142,143,144,145,146,147,148,149,150,151].

### 3.2. Overview of the Included Studies

Because studies frequently evaluated more than one restoration type and reported on more than one outcome domain, studies could be coded under multiple categories in the restoration-type and outcome-domain classifications below; consequently, the sum of records across these categories exceeds the total number of included studies. In contrast, each study was assigned to a single material family.

The 111 included studies were mainly experimental. In vitro investigations accounted for the large majority of the evidence base (105 studies). Clinical evidence was comparatively scarce and comprised five non-randomised clinical studies [69,78,105,118,119] and a single randomised controlled trial [56], together representing only six studies (approximately 5% of the corpus). Two of these six clinical reports [118,119] derive from the same registered clinical trial, reporting colour stability and wear outcomes, respectively, from overlapping patient cohorts at the same institution. The six clinical reports therefore represent five unique clinical studies.

Table 1 presents the distribution of the 111 included studies by material family.

Four material categories were identified. Printable resin and resin-composite materials were the most frequently investigated (57 studies), followed by zirconia-based ceramics (50 studies). Two studies directly compared additively manufactured zirconia and resin materials; both arms met the eligibility criteria, and they are reported separately (Supplementary Table S3). The remaining two studies evaluated materials outside these dominant families: a feldspar glass–ceramic and a gradient zirconia/resin-infiltrated composite. Together, these categories account for all 111 included studies (57 + 50 + 2 + 2 = 111).

**Figure 2:**
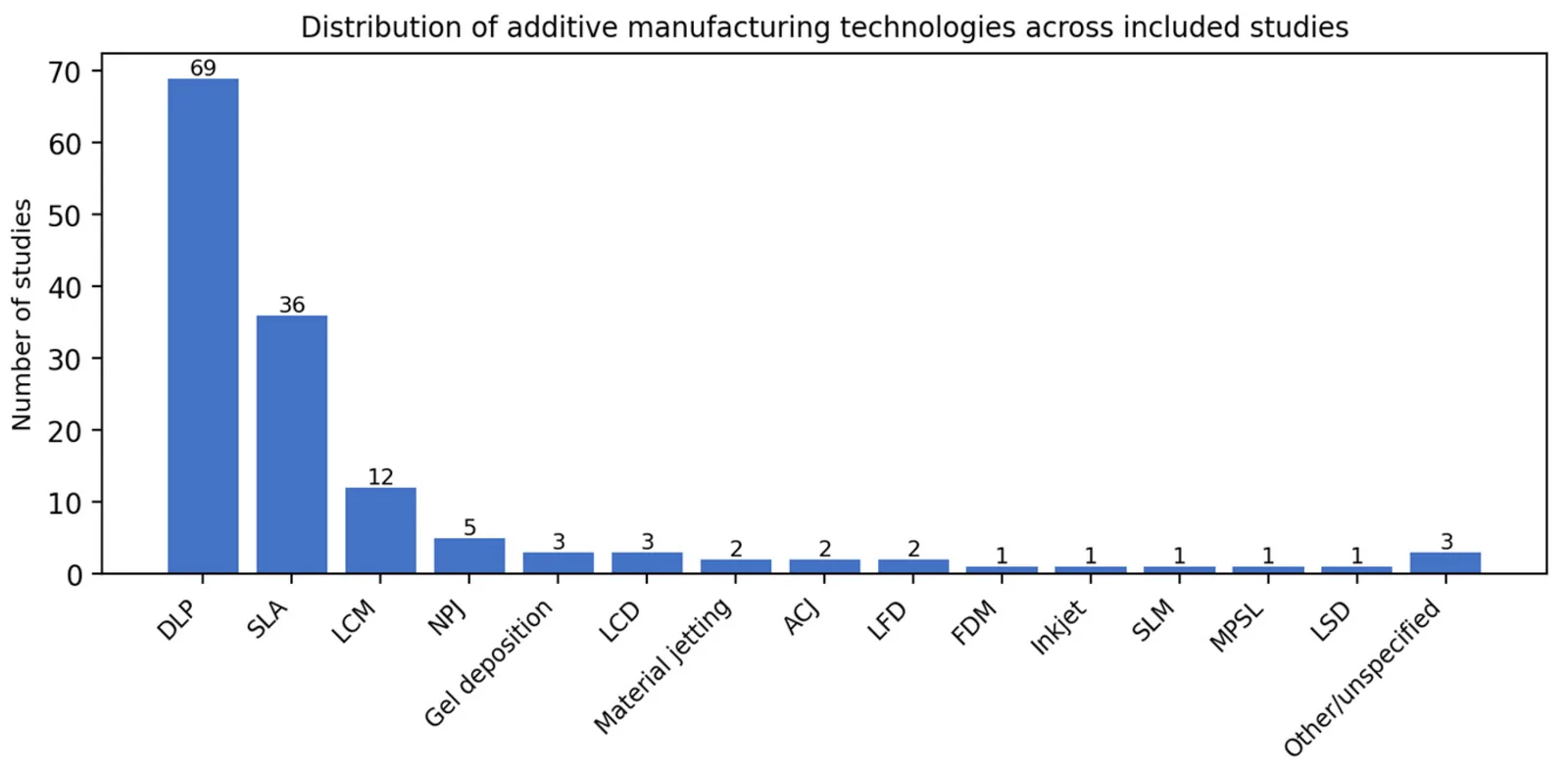
Additive manufacturing technologies represented across included studies. Note: Some studies report more than one technology (e.g., multi-technology comparative studies).

Figure 2 presents the distribution of additive manufacturing technologies represented across the included studies.

A broad spectrum of additive manufacturing technologies was represented, but the distribution was markedly skewed. Vat-photopolymerization approaches predominated, with digital light processing (DLP) the most commonly reported technology, followed by stereolithography (SLA) and lithography-based ceramic manufacturing (LCM) [49,52,57]. A technologically diverse tail comprised droplet- and jetting-based methods [22,48,55,56,71,73], fused deposition modelling (FDM) [44], selective laser melting (SLM) [69], gel-deposition/”self-glazed” zirconia processes [78,79,87], layerwise slurry deposition (LSD-print) [73], mask-projection stereolithography (MPSL) [74], and several vat-photopolymerization variants including tilting SLA (TSLA), masked SLA (MSLA), digital press stereolithography (DPS), and liquid-crystal-display (LCD) and low-force-display (LFD) printing [107,117,128,143,144]. Nearly all comparative studies benchmarked additively manufactured restorations against subtractively milled counterparts.

Table 2 presents the distribution of the included studies by restoration type.

With respect to indication, single crowns were the dominant restoration type (73 studies), followed by fixed dental prostheses (FDPs; 18 studies), veneers and occlusal table-tops (11 studies), endocrowns (10 studies), overlays (3 studies) [129,133,151], and a single onlay study [135]. No study exclusively addressed an inlay restoration; the one inlay-related design identified was an inlay-retained fixed dental prosthesis, classified under FDPs.

**Figure 3:**
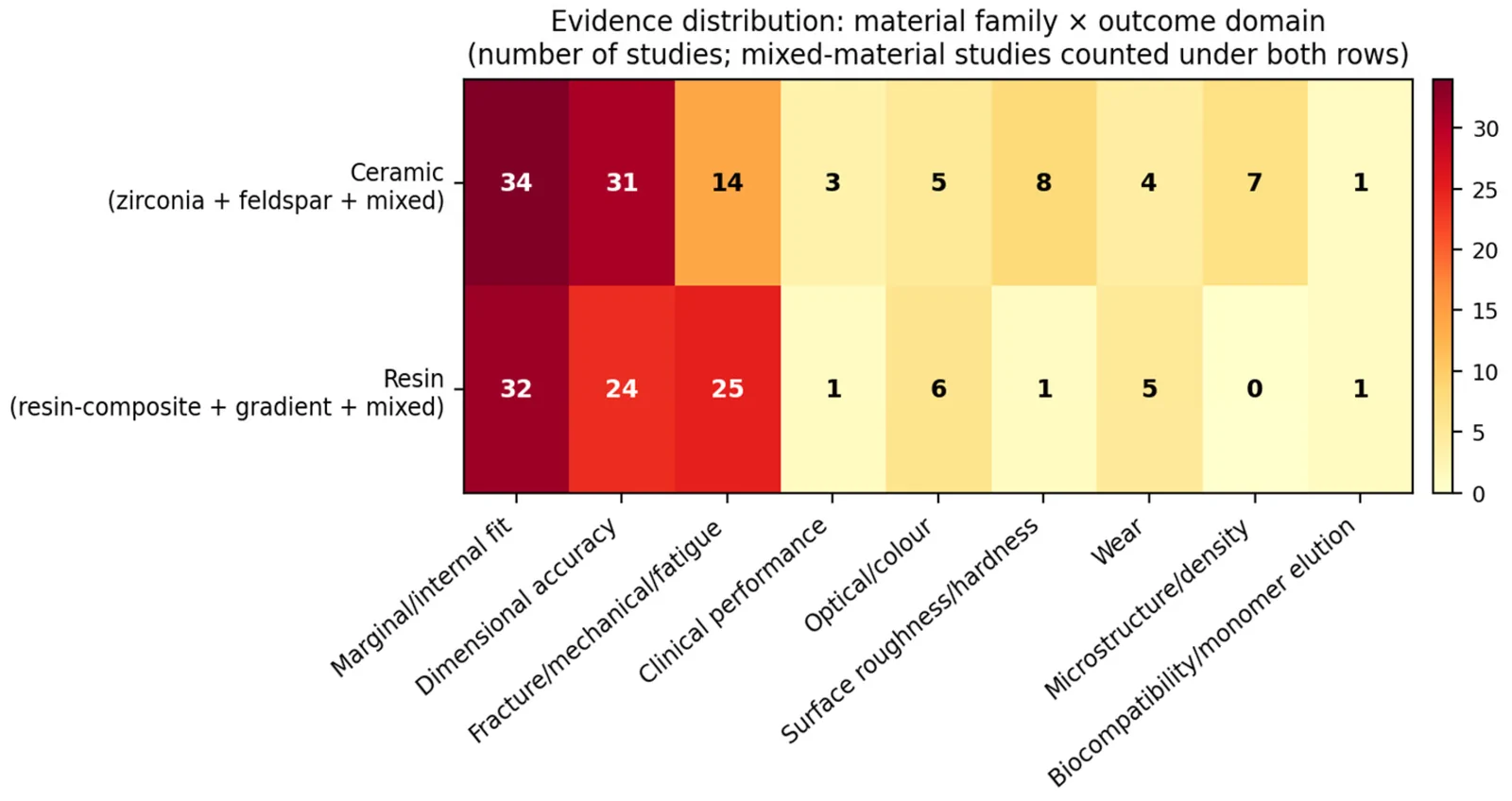
Evidence distribution heatmap (material family × outcome domain).

Figure 3 presents the distribution of evaluated outcome domains across the two dominant material families.

The evaluated outcomes clustered into recurring domains. Marginal and internal fit/adaptation was the most frequently assessed outcome (67 studies), closely followed by dimensional accuracy expressed as trueness and precision (55 studies) and by fracture resistance, fatigue, and related mechanical behaviour (36 studies). Less frequently assessed domains included clinical performance (12 studies), surface roughness and hardness (11 studies), optical and colour properties (11 studies), wear (8 studies), microstructure and density (7 studies), and biocompatibility or residual-monomer elution (2 studies). A consistent cross-cutting theme, examined within both material families, was the influence of processing parameters—most notably build angle/orientation, layer thickness, post-polymerisation or sintering protocol, cement-space setting, and pixel offset—on the accuracy and performance of the resulting restorations.

### 3.3. Additively Manufactured Zirconia Ceramic Restorations

Across 49 studies, dimensional accuracy and fit were the dominant outcomes. Milled zirconia generally achieved equal or higher trueness, while printed crowns frequently matched or exceeded it in precision, with both routes reported as within clinically acceptable limits by the primary studies [47,57,67,68,86]; jetting-based technologies (NPJ, ACJ) reported trueness and margin quality exceeding milling [48,55,71]; jetting-based technologies (NPJ, ACJ) reported trueness and margin quality exceeding milling [48,55,71]; among vat-photopolymerization methods, LCM and SLA tended to outperform DLP [84]. Printed crowns and FDPs commonly showed larger marginal/internal gaps than milled controls yet remained within the thresholds defined as acceptable by the respective primary studies, including after ageing [46,50,80], with support-frame and margin design modifying fit [59,63]. Fracture resistance was comparable to milled zirconia and above physiological loads after fatigue [49,61,77,87], though printed surfaces were rougher and contained more internal voids before finishing [49,70]. Optical, wear, and clinical data were sparse but reported acceptable short-term performance [56,69,76,77,78].

### 3.4. Additively Manufactured Resin Restorations

Across 60 studies, printed resins frequently matched or exceeded milled hybrid/ceramic materials in dimensional accuracy and marginal adaptation [99,120,132,141], with preparation and design factors (margin geometry, pulp-chamber depth, cement-space, scanner–printer combination) strongly influencing fit [92,97,116,130,131]. Printed restorations reliably survived long-term cyclic fatigue with frequently repairable failures, but showed lower absolute fracture resistance than milled and ceramic comparators and were sensitive to ageing and thickness [90,100,112,113,135]. Colour instability was a recurring limitation, with several in vitro and clinical studies reporting discolouration exceeding acceptability thresholds and recommending interim or short-term use [118,122,149]; residual-monomer elution exceeded that of milled comparators but stayed below cytotoxic thresholds [106]. Clinical evidence (24-month follow-up) reported acceptable function but progressive discolouration and occlusal wear [105,118,119].

### 3.5. Other Materials

Two studies addressed materials outside the two dominant families. A feldspar glass–ceramic fabricated by layerwise slurry deposition reproduced nominal crown and veneer geometry well in the as-printed state (>95% of the surface within 100 µm) but developed deviations up to 300 µm after firing due to viscous-flow deformation, indicating that shape retention during firing requires further development before adequate fit is achievable [75]. A DLP-printed gradient zirconia/resin-infiltrated composite crown closely matched its design model and showed good biocompatibility, supporting the feasibility of gradient ceramic-polymer crowns as a proof of concept [124].

### 3.6. Influence of Processing Parameters Across Materials

A prominent cross-cutting finding, recurring throughout both material families, was that fabrication and performance outcomes were strongly influenced by processing parameters rather than by additive manufacturing per se. Build angle and build orientation repeatedly emerged as significant determinants of trueness, marginal quality, and intaglio accuracy for both printed zirconia and printed resins, with material- and geometry-specific optima [73,83,103,127,145]. Layer thickness influenced accuracy, with finer layers generally improving trueness [83]. Post-processing protocols—sintering temperature and schedule for ceramics, and post-polymerisation time, medium, temperature, and inert atmosphere for resins—significantly modulated mechanical, dimensional, and surface properties [42,95,146]. Digitally controlled design variables, including cement-space setting, margin/finish-line design, pulp-chamber depth for endocrowns, and pixel offset, exerted effects that frequently equalled or exceeded those of the fabrication route itself [81,97,130,138]. Collectively, these findings indicate that optimisation of the AM workflow is a prerequisite for achieving clinically acceptable definitive restorations, and that many discrepancies attributed to printing can be mitigated through parameter control.

## 4. Discussion

This scoping review mapped the available evidence on additively manufactured resin- and ceramic-based materials for definitive tooth-supported crowns and fixed dental prostheses. The 111 included studies reveal a field expanding rapidly but unevenly: the literature is dominated by in vitro investigation, concentrated on a narrow set of materials and vat-photopolymerization technologies, and directed overwhelmingly at single crowns. The principal finding of this map is therefore twofold—additive manufacturing of definitive restorations is technically feasible and, across several outcome domains, associated with outcomes reported as comparable to subtractive milling, yet the clinical evidence base required to support routine definitive use remains significantly thin.

The distribution of study designs is the clearest signal to emerge. With 105 of 111 studies being laboratory investigations and only six clinical reports, representing five unique clinical studies (two reports [118,119] originate from the same registered trial, of which a single one was a randomised controlled trial [56], the field rests almost entirely on in vitro data. This imbalance is characteristic of an emerging technology, where bench testing necessarily precedes clinical translation, but it also defines the central evidence gap. In vitro accuracy, fit, and fracture data are informative proxies, yet they cannot capture the intraoral variables—moisture, thermal and pH cycling, parafunction, and the biological response of the periodontium—that ultimately determine clinical longevity. The available clinical reports, though encouraging within short observation windows [56,69,78,105], are limited by small samples, follow-up rarely exceeding 24 months, and heterogeneous outcome reporting. The evidence, in short, supports feasibility far more strongly than it supports durability.

Within the ceramic literature, printed zirconia emerged as the most thoroughly characterised material, and the pattern across studies is coherent. Subtractive milling generally retained an advantage in trueness in the studies that directly compared the two techniques, whereas printed crowns frequently matched or exceeded it in precision; both routes were reported to produce restorations within the clinically accepted thresholds defined in the primary studies [47,57,67,68,86]. This distinction between trueness and precision is clinically meaningful: high precision indicates that printing produces consistent, reproducible restorations even where a systematic offset from the reference exists, and such offsets are frequently correctable through parameter calibration. Notably, jetting-based technologies and self-glazed processes reported accuracy, fit, and even fracture behaviour rivalling or surpassing milling [48,55,71,87], suggesting that the performance ceiling of additive zirconia is not yet defined by the vat-photopolymerization methods that currently dominate. The finding that printed zirconia can achieve fracture resistance comparable to milled controls and well above physiological loads [49,61,77,87] is reassuring, though the greater surface roughness and internal porosity observed before finishing [49,70] underline that post-processing is integral rather than incidental to acceptable performance.

The resin literature presents a more ambivalent picture, and one where the gap between laboratory promises and clinical caution is widest. Printed resins repeatedly matched or exceeded milled hybrid materials in accuracy and marginal adaptation [99,120,132,141] and reliably survived long-term cyclic fatigue with the added, clinically attractive property of reparability [100,112,141]. However, their lower absolute fracture resistance relative to milled ceramics and their sensitivity to ageing and restoration thickness [90,113,135] temper enthusiasm for load-bearing applications, particularly at reduced thicknesses. The most consistent limitation was colour instability: multiple in vitro and clinical studies documented discolouration exceeding acceptability thresholds over time, prompting several authors to recommend interim or short-term use despite manufacturers’ designation of these materials as definitive [118,122,151]. This discrepancy between commercial labelling and empirical performance is a recurring theme deserving explicit attention, as it directly affects clinical decision-making and patient expectations. The elevated residual-monomer elution reported for some printable resins, albeit below cytotoxic thresholds [106], similarly warrants continued scrutiny given the limited biocompatibility data overall—only two studies addressed this domain.

A cross-cutting theme, and arguably the most clinically actionable finding of this review, is the extent to which outcomes depend on processing parameters rather than on additive manufacturing as such. Build angle and orientation, layer thickness, and post-processing protocols repeatedly exerted effects on accuracy, fit, and mechanical behaviour that equalled or exceeded those attributable to the fabrication route itself [42,73,83,145]. This dependency has a double implication. On the one hand, it explains much of the heterogeneity across studies and cautions against generalising results obtained under one set of parameters to differently configured workflows. On the other, it means that many discrepancies attributed to printing are correctable, and that the standardisation of printing and post-processing protocols is a prerequisite for reliable clinical outcomes. The current absence of consensus protocols is thus not merely a research inconvenience but a barrier to clinical adoption.

These findings should be interpreted in light of the scope and limitations of the review itself. As a scoping review, its purpose was to map the breadth and distribution of evidence rather than to appraise its quality; consequently, no formal risk-of-bias assessment or quantitative synthesis was undertaken, and the descriptive conclusions drawn here are correspondingly tentative [39,40,41]. In addition, the review protocol was registered after the literature search and screening had already been completed, and should therefore be regarded as retrospectively rather than prospectively registered; while the eligibility criteria and search strategy were fixed in advance of the search, this timing limits the extent to which selective outcome reporting during screening can be excluded. The considerable heterogeneity in study designs, outcome measures, testing protocols, and reporting that precluded meta-analysis also constrains direct comparison between studies within this map. Restricting eligibility to tooth-supported crowns and FDPs—excluding implant-supported and other indications—may have omitted relevant evidence. In addition, the search was limited to English-language publications, which may have introduced language bias and led to the omission of relevant studies reported in other languages. The fast pace of publication in this field also means that new studies will continue to appear beyond the search date. Finally, because the primary literature is weighted so heavily toward a small number of materials and technologies, the map inevitably reflects where research attention has concentrated rather than the full range of clinically available options.

Taken together, the evidence maps a technology on the threshold of clinical translation. Additive manufacturing of definitive restorations has convincingly demonstrated in vitro feasibility and, in several domains, outcomes reported as comparable to milling, but the current literature makes clear that the transition to routine clinical use awaits three developments: long-term randomised clinical trials, standardised printing and post-processing protocols, and resolution of the material-specific limitations—colour stability and wear for resins, surface finishing and multi-unit performance for ceramics—that presently distinguish laboratory promise from clinical readiness. Future research directed at these priorities, rather than at further replication of favourable in vitro accuracy data, would most efficiently advance the field.

## 5. Conclusions

This scoping review mapped the current evidence on additively manufactured resin- and ceramic-based materials for definitive tooth-supported crowns and fixed dental prostheses. The field is expanding rapidly but unevenly, with the evidence concentrated on single crowns, a narrow range of materials, and vat-photopolymerization technologies. Across dimensional accuracy, marginal and internal fit, and fracture resistance, additively manufactured restorations were frequently reported as comparable to subtractively milled controls and generally within the clinically accepted thresholds defined by the primary studies, with the reported outcomes most consistent for printed zirconia and printed resins more often limited by lower strength and colour instability. Outcomes were strongly governed by processing parameters, making workflow standardisation a prerequisite for reproducible results. Critically, with only six clinical studies including one randomised controlled trial, the evidence supports technical feasibility far more than long-term clinical durability. Future research should prioritise long-term randomised clinical trials, standardised protocols, and resolution of material-specific limitations to enable routine clinical translation.

## Figures and Tables

**Table 1:** Distribution of included studies by material family.

Material Family	Studies (n)	Percentage
Resin/resin-composite	57	51.4%
Zirconia ceramic	50	45.0%
Mixed-material (zirconia + resin)	2	1.8%
Feldspar glass–ceramic	1	0.9%
Gradient zirconia/resin-composite	1	0.9%
Total	111	100%

**Table 2:** Distribution of included studies by restoration type (studies may address more than one type).

Restoration Type	Studies (n)
Single crown	73
Fixed dental prosthesis (FDP)	18
Veneer/occlusal table-top	11
Endocrown	10
Overlay	3
Onlay	1

Note: Studies may address more than one restoration type. One study [104] reported no discrete restoration type and is excluded from this table (110 of 111 studies classified).

## Data Availability

No new data were created or analysed in this study. Data sharing is not applicable to this article.

## Supplementary Materials

The following supporting information can be downloaded at https://www.mdpi.com/article/10.3390/ma19173728/s1: Table S1: Study characteristics and extracted data for additively manufactured ceramic-based restorations; Table S2: Study characteristics and extracted data for additively manufactured resin-based restorations; Table S3: Study characteristics and extracted data for studies directly comparing additively manufactured zirconia and resin materials. File S1: Scoping Reviews (PRISMA-ScR) Checklist.

## Abbreviations

The following abbreviations are used in this manuscript:

3DP	Three-dimensional printing
3Y-TZP	3 mol% yttria-stabilised tetragonal zirconia polycrystal
ACJ	Advanced customised jetting
AM	Additive manufacturing
CAD/CAM	Computer-aided design/computer-aided manufacturing
DLP	Digital light processing
DPS	Digital press stereolithography
FDM	Fused deposition modelling
FDP	Fixed dental prosthesis
JBI	Joanna Briggs Institute
LCD	Liquid-crystal display
LCM	Lithography-based ceramic manufacturing
LFD	Low-force display
LSD	Layerwise slurry deposition
MPSL	Mask-projection stereolithography
MSLA	Masked stereolithography
NC	Numerical control
NPJ	Nanoparticle jetting
OSF	Open Science Framework
PCC	Population–Concept–Context
PRISMA-ScR	Preferred Reporting Items for Systematic Reviews and Meta-Analyses extension for Scoping Reviews
PSZ	Partially stabilised zirconia
SLA	Stereolithography
SLM	Selective laser melting
SLS	Selective laser sintering
TSLA	Tilting stereolithography
Y-TZP	Yttria-stabilised tetragonal zirconia polycrystal

## Appendix A. Detailed Search Strategies

The detailed database-specific strategies were as follows. In the PubMed (MEDLINE) strategy, terms designated [MeSH Terms] were searched as controlled-vocabulary Medical Subject Headings, while all remaining terms were entered as free-text keywords without a field tag and were therefore processed through PubMed’s Automatic Term Mapping and searched across all fields. In Embase, free-text terms were restricted to title and abstract (:ti,ab) and controlled terms were exploded (/exp); in Scopus, all terms were searched within TITLE-ABS-KEY. The asterisk (*) denotes truncation to capture spelling and plural variants.

- PubMed (MEDLINE):

(((((((((3d printing[MeSH Terms]) OR (3d printing)) OR (3d printed)) OR (additive* manufactur*)) OR (rapid prototyp*)) OR (stereolithography[MeSH Terms])) OR (stereolithograph*)) OR (polyjet printing)) OR (fused deposition modeling)) OR (three dimensional print*)

AND

((((((((((((((((((dental restorations, permanent[MeSH Terms]) OR (dental restoration, permanent[MeSH Terms])) OR (denture, fixed partial[MeSH Terms])) OR (fixed partial denture[MeSH Terms])) OR (dental restoration*)) OR (dental crown*)) OR (implant crown*)) OR (dental prosthesis, implant supported[MeSH Terms])) OR (fixed dental prosthes*)) OR (fixed restoration*)) OR (FDP)) OR (fixed partial denture*)) OR (inlay*)) OR (veneer*)) OR (onlay*)) OR (overlay*)) OR (endocrown*)) OR (partial restoration*)) OR (partial crown*)

- Embase:

(‘3d printing’/exp OR ‘3d printing’:ti,ab OR ‘3d printed’:ti,ab OR ‘additive manufacturing’:ti,ab OR ‘rapid prototyping’:ti,ab OR ‘stereolithography’/exp OR stereolithograph*:ti,ab OR ‘polyjet printing’:ti,ab OR ‘fused deposition modeling’:ti,ab OR ‘three dimensional print*’:ti,ab)

AND

(‘dental restoration’/exp OR ‘permanent dental restoration’/exp OR ‘fixed partial denture’/exp OR ‘implant supported dental prosthesis’/exp OR ‘dental crown’/exp OR ‘dental restoration*’:ti,ab OR ‘dental crown*’:ti,ab OR ‘implant crown*’:ti,ab OR ‘fixeddental prosthes*’:ti,ab OR ‘fixed restoration*’:ti,ab OR FDP:ti,ab OR ‘fixed partial denture*’:ti,ab OR inlay*:ti,ab OR veneer*:ti,ab OR onlay*:ti,ab OR overlay*:ti,ab OR endocrown*:ti,ab OR ‘partial restoration*’:ti,ab OR ‘partial crown*’:ti,ab)

- Scopus:

TITLE-ABS-KEY(“3D printing” OR “3D printed” OR “additive manufacturing” OR “rapid prototyping” OR stereolithograph* OR “polyjet printing” OR “fused deposition modeling” OR “three dimensional print*”)

AND

(TITLE-ABS-KEY(“dental restoration*” OR “dental crown*” OR “implant crown*” OR “fixed dental prosthes*” OR “fixed restoration*” OR FDP OR “fixed partial denture*” OR inlay* OR veneer* OR onlay* OR overlay* OR endocrown* OR “partial restoration*” OR “partial crown*”))

## References

[1] Tapie L., Lebon N., Mawussi B., Fron-Chabouis H., Duret F., Attal J.P. (2015). Understanding dental CAD/CAM for restorations—Accuracy from a mechanical engineering viewpoint. Int. J. Comput. Dent..

[2] Hassanpour M., Narongdej P., Alterman N., Moghtadernejad S., Barjasteh E. (2024). Effects of Post-Processing Parameters on 3D-Printed Dental Appliances: A Review. Polymers.

[3] Lee W.S., Lee D.H., Lee K.B. (2017). Evaluation of internal fit of interim crown fabricated with CAD/CAM milling and 3D printing system. J. Adv. Prosthodont..

[4] Zhang Y., Lawn B.R. (2019). Evaluating dental zirconia. Dent. Mater..

[5] Rodrigues C.D.S., Aurélio I.L., Kaizer M.D.R., Zhang Y., May L.G. (2019). Do thermal treatments affect the mechanical behavior of porcelain-veneered zirconia? A systematic review and meta-analysis. Dent. Mater..

[6] Christensen G.J. (2007). Choosing an all-ceramic restorative material: Porcelain-fused-to-metal or zirconia-based?. J. Am. Dent. Assoc..

[7] Maroulakos G., Thompson G.A., Kontogiorgos E.D. (2019). Effect of cement type on the clinical performance and complications of zirconia and lithium disilicate tooth-supported crowns: A systematic review. J. Prosthet. Dent..

[8] Brandt S., Winter A., Lauer H.C., Kollmar F., Portscher-Kim S.J., Romanos G.E. (2019). IPS e.max for All-Ceramic Restorations: Clinical Survival and Success Rates of Full-Coverage Crowns and Fixed Partial Dentures. Materials.

[9] Spitznagel F.A., Boldt J., Gierthmuehlen P.C. (2018). CAD/CAM Ceramic Restorative Materials for Natural Teeth. J. Dent. Res..

[10] Daher R., Ardu S., di Bella E., Krejci I., Duc O. (2024). Efficiency of 3D printed composite resin restorations compared with subtractive materials: Evaluation of fatigue behavior, cost, and time of production. J. Prosthet. Dent..

[11] Ioannidis A., Mühlemann S., Özcan M., Hüsler J., Hämmerle C.H.F., Benic G.I. (2019). Ultra-thin occlusal veneers bonded to enamel and made of ceramic or hybrid materials exhibit load-bearing capacities not different from conventional restorations. J. Mech. Behav. Biomed. Mater..

[12] Rezaie F., Farshbaf M., Dahri M., Masjedi M., Maleki R., Amini F., Wirth J., Moharamzadeh K., Weber F.E., Tayebi L. (2023). 3D Printing of Dental Prostheses: Current and Emerging Applications. J. Compos. Sci..

[13] Pot G.J., Van Overschelde P.A., Keulemans F., Kleverlaan C.J., Tribst J.P.M. (2024). Mechanical Properties of Additive-Manufactured Composite-Based Resins for Permanent Indirect Restorations: A Scoping Review. Materials.

[14] Kirby S., Pesun I., Nowakowski A., França R. (2024). Effect of Different Post-Curing Methods on the Degree of Conversion of 3D-Printed Resin for Models in Dentistry. Polymers.

[15] Mandurino M., Cortili S., Coccoluto L., Greco K., Cantatore G., Gherlone E.F., Vichi A., Paolone G. (2025). Mechanical Properties of 3D Printed vs. Subtractively Manufactured Composite Resins for Permanent Restorations: A Systematic Review. Materials.

[16] Sim M.Y., Park J.B., Kim D.Y., Kim H.Y., Park J.M. (2024). Dimensional accuracy and surface characteristics of complete-arch cast manufactured by six 3D printers. Heliyon.

[17] Edgar J., Tint S. (2015). Additive manufacturing technologies: 3D printing, rapid prototyping, and direct digital manufacturing, 2nd Edition. Johns. Matthey Technol. Rev..

[18] Ngo T.D., Kashani A., Imbalzano G., Nguyen K.T.Q., Hui D. (2018). Additive manufacturing (3D printing): A review of materials, methods, applications and challenges. Compos. Part B Eng..

[19] Alghauli M., Alqutaibi A.Y., Wille S., Kern M. (2024). 3D-printed versus conventionally milled zirconia for dental clinical applications: Trueness, precision, accuracy, biological and esthetic aspects. J. Dent..

[20] Camargo B., Willems E., Jacobs W., Van Landuyt K., Peumans M., Zhang F., Vleugels J., Van Meerbeek B. (2022). 3D printing and milling accuracy influence full-contour zirconia crown adaptation. Dent. Mater..

[21] Andjela L., Abdurahmanovich V.M., Vladimirovna S.N., Mikhailovna G.I., Yurievich D.D., Alekseevna M.Y. (2022). A review on Vat Photopolymerization 3D-printing processes for dental application. Dent. Mater..

[22] Su G., Zhang Y., Jin C., Zhang Q., Lu J., Liu Z., Wang Q., Zhang X., Ma J. (2023). 3D printed zirconia used as dental materials: A critical review. J. Biol. Eng..

[23] Miyazaki T., Hotta Y., Kunii J., Kuriyama S., Tamaki Y. (2009). A review of dental CAD/CAM: Current status and future perspectives from 20 years of experience. Dent. Mater. J..

[24] Cai H., Xu X., Lu X., Zhao M., Jia Q., Jiang H.B., Kwon J.S. (2023). Dental Materials Applied to 3D and 4D Printing Technologies: A Review. Polymers.

[25] Huang G., Wu L., Hu J., Zhou X., He F., Wan L., Pan S.T. (2022). Main Applications and Recent Research Progresses of Additive Manufacturing in Dentistry. BioMed Res. Int..

[26] Çakmak Alp G., Cuellar A., Donmez M.B., Schimmel M., Abou-Ayash S., Lu W.E., Yilmaz B. (2021). Effect of printing layer thickness on the trueness and margin quality of 3D-printed interim dental crowns. Appl. Sci..

[27] Çakmak Alp G., Cuellar A., Donmez M.B., Abou-Ayash S., Lu W.E., Schimmel M., Yilmaz B. (2024). Effect of printing layer thickness on the trueness of 3-unit interim fixed partial dentures. J. Prosthet. Dent..

[28] Li H., Song L., Sun J., Ma J., Shen Z. (2019). Dental ceramic prostheses by stereolithography-based additive manufacturing: Potentials and challenges. Adv. Appl. Ceram..

[29] Dehurtevent M., Robberecht L., Hornez J.C., Thuault A., Deveaux E., Béhin P. (2017). Stereolithography: A new method for processing dental ceramics by additive computer-aided manufacturing. Dent. Mater..

[30] Ferrage L., Bertrand G., Lenormand P., Grossin D., Ben-Nissan B. (2017). A review of the additive manufacturing (3DP) of bioceramics: Alumina, zirconia (PSZ) and hydroxyapatite. J. Aust. Ceram. Soc..

[31] Khanlar L.N., Salazar Rios A., Tahmaseb A., Zandinejad A. (2021). Additive Manufacturing of Zirconia Ceramic and Its Application in Clinical Dentistry: A Review. Dent. J..

[32] Ramezani M., Dommati H., Wang J.C., Pasang T., Lee C. (2023). Tribological characterization of alumina ceramic manufactured by solvent-based slurry stereolithography. J. Mater. Eng. Perform..

[33] Zimmermann M., Ender A., Egli G., Özcan M., Mehl A. (2019). Fracture load of CAD/CAM-fabricated and 3D-printed composite crowns as a function of material thickness. Clin. Oral Investig..

[34] Alamoush R.A., Silikas N., Salim N.A., Al-Nasrawi S., Satterthwaite J.D. (2018). Effect of the Composition of CAD/CAM Composite Blocks on Mechanical Properties. BioMed Res. Int..

[35] Sedrez-Porto J.A., Rosa W.L., da Silva A.F., Münchow E.A., Pereira-Cenci T. (2016). Endocrown restorations: A systematic review and meta-analysis. J. Dent..

[36] Bayarsaikhan E., Lim J.H., Shin S.H., Park K.H., Park Y.B., Lee J.H., Kim J.E. (2021). Effects of Postcuring Temperature on the Mechanical Properties and Biocompatibility of Three-Dimensional Printed Dental Resin Material. Polymers.

[37] Baytur S., Diken Turksayar A.A. (2025). Effects of post-polymerization conditions on color properties, surface roughness, and flexural strength of 3D-printed permanent resin material after thermal aging. J. Prosthodont..

[38] Tahayeri A., Morgan M., Fugolin A.P., Bompolaki D., Athirasala A., Pfeifer C.S., Ferracane J.L., Bertassoni L.E. (2018). 3D printed versus conventionally cured provisional crown and bridge dental materials. Dent. Mater..

[39] Page M.J., Moher D., Bossuyt P.M., Boutron I., Hoffmann T.C., Mulrow C.D., Shamseer L., Tetzlaff J.M., Akl E.A., Brennan S.E. (2021). PRISMA 2020 explanation and elaboration: Updated guidance and exemplars for reporting systematic reviews. BMJ.

[40] Peters M., Godfrey C., Mcinerney P., Tricco A., Khalil H., Aromataris E., Munn Z. (2020). Chapter 11: Scoping Reviews. JBI Manual for Evidence Synthesis.

[41] Tricco A.C., Lillie E., Zarin W., O’Brien K.K., Colquhoun H., Levac D., Moher D., Peters M.D., Horsley T., Weeks L. (2018). PRISMA Extension for Scoping Reviews (PRISMA-ScR): Checklist and Explanation. Ann. Intern. Med..

[42] Su J.J., Li X.Y., Liu Z.M., Wu J.M., Huang C., Yao C.M., Zhang X.Y., Wang X.M., Shi Y.S. (2025). Mechanical and biological properties of zirconia crowns prepared by digital light processing. J. Am. Ceram. Soc..

[43] Kalman L., Tribst J.P.M. (2024). Quality Assessment and Comparison of 3D-Printed and Milled Zirconia Anterior Crowns and Veneers: In Vitro Pilot Study. Eur. J. Gen. Dent..

[44] Kumar R.G., Rengasamy V., Karthik S.P., Kumar S.A., Kanna M.K. (2025). Trueness, precision, fit of FDM printed vs multilayered milled dental restorations: An in vitro study. Eur. J. Prosthodont. Restor. Dent..

[45] Refaie A., Fouda A., Bourauel C., Singer L. (2023). Marginal gap and internal fit of 3D printed versus milled monolithic zirconia crowns. BMC Oral Health.

[46] Hassan R.M., AboELHassan R.G., Azer A.S. (2025). Analysis of the marginal gap and internal fit accuracy of 3D printed zirconia crowns using the triple scan protocol. J. Prosthet. Dent..

[47] Refaie A., Bourauel C., Elshazly T., Evers-Dietze B., Alhotan A., Aldesoki M. (2024). Trueness and precision of digital light processing fabricated 3D printed monolithic zirconia crowns. J. Dent..

[48] Zhu H., Zhou Y., Jiang J., Wang Y., He F. (2025). Accuracy and margin quality of advanced 3D printed monolithic zirconia crowns. J. Prosthet. Dent..

[49] Hassan R.M., Ibrahim Y., AboELHassan R.G., Azer A.S. (2025). Evaluation of fracture resistance and surface characteristics in monolithic zirconia: A comparative analysis of 3D printing and milling techniques. BMC Oral Health.

[50] Elsayed M.S., El-Kouedi A.Y., Shokry T.E. (2025). Effect of aging on the marginal fit of milled and printed zirconia crowns: An in-vitro study. BMC Oral Health.

[51] Wang W., Yu H., Liu Y., Jiang X., Gao B. (2019). Trueness analysis of zirconia crowns fabricated with 3-dimensional printing. J. Prosthet. Dent..

[52] Refaie A., Bourauel C., Fouda A.M., Keilig L., Singer L. (2023). The effect of cyclic loading on the fracture resistance of 3D-printed and CAD/CAM milled zirconia crowns-an in vitro study. Clin. Oral Investig..

[53] Li R., Xu T., Wang Y., Sun Y. (2023). Accuracy of zirconia crowns manufactured by stereolithography with an occlusal full-supporting structure: An in vitro study. J. Prosthet. Dent..

[54] Lüchtenborg J., Willems E., Zhang F., Wesemann C., Weiss F., Nold J., Sun J., Sandra F., Bai J., Reveron H. (2022). Accuracy of additively manufactured zirconia four-unit fixed dental prostheses fabricated by stereolithography, digital light processing and material jetting compared with subtractive manufacturing. Dent. Mater..

[55] Wang Y., Zhou Y., Zhu H., Jiang J., He F. (2025). Accuracy, fit, and marginal quality of advanced additively manufactured and milled zirconia 3-unit fixed dental prostheses. J. Prosthet. Dent..

[56] Cai F., Wang Y., Jiang J., Wu W., Lu H., Ye T., Zhou Y., He F. (2025). Clinical efficacy of a novel 3D-printed zirconia single crown: A randomized controlled trial. J. Dent..

[57] Lerner H., Nagy K., Pranno N., Zarone F., Admakin O., Mangano F. (2021). Trueness and precision of 3D-printed versus milled monolithic zirconia crowns: An in vitro study. J. Dent..

[58] Lee H.B., Noh M.J., Bae E.J., Lee W.S., Kim J.H. (2024). Accuracy of zirconia crown manufactured using stereolithography and digital light processing. J. Dent..

[59] Li R., Chen H., Wang Y., Sun Y. (2021). Performance of stereolithography and milling in fabricating monolithic zirconia crowns with different finish line designs. J. Mech. Behav. Biomed. Mater..

[60] Dagistan S., Toksoy D., Önöral Ö., Diken Turksayar A.A. (2025). Effect of different additive manufacturing technologies on the fracture load of 3-unit monolithic zirconia fixed partial dentures: In vitro mechanical evaluation and energy-dispersive spectroscopy analysis. J. Prosthet. Dent..

[61] Zhai Z., Qian C., Jiao T., Sun J. (2025). In vitro fracture and fatigue resistance of monolithic zirconia crowns fabricated by stereolithography. J. Prosthet. Dent..

[62] Rues S., Crocoll J., Hetzler S., Rossipal J., Rammelsberg P., Zenthöfer A. (2025). Fracture Resistance of 3-Unit Zirconia Fixed Dental Prostheses Differing in Wall Thickness Fabricated by Either 3D-Printing or Milling. J. Funct. Biomater..

[63] Kostunov J., Crocoll J., Hetzler S., Rammelsberg P., Zeiß J., Zenthöfer A., Rues S. (2026). Fit of Three-Unit Posterior Fixed Dental Prostheses Made from Tetragonal Zirconia Polycrystal by 3D Printing and Milling. Materials.

[64] Li B., Jiang Q., Meng D. (2023). Evaluation of the trueness and adaptation of zirconia crowns fabricated with stereolithography. Dent. Mater. J..

[65] Li R., Wang Y., Hu M., Wang Y., Xv Y., Liu Y., Sun Y. (2019). Strength and Adaptation of Stereolithography-Fabricated Zirconia Dental Crowns: An In Vitro Study. Int. J. Prosthodont..

[66] Toksoy D., Önöral Ö. (2024). Influence of glazing and aging on the marginal, axial, axio-occlusal, and occlusal fit of 3-unit monolithic zirconia restorations fabricated using additive and subtractive techniques. J. Prosthet. Dent..

[67] Cho S.M., Park J.M., Ioannidis A., Kim R.J.Y., Chung H.M. (2025). Comparative analysis of fit, mechanical properties, and surface characteristics in subtractive- and additive-manufactured zirconia crowns. BMC Oral Health.

[68] Abualsaud R., Alalawi H. (2022). Fit, Precision, and Trueness of 3D-Printed Zirconia Crowns Compared to Milled Counterparts. Dent. J..

[69] Kao C.T., Liu S.H., Kao C.Y., Huang T.H. (2023). Clinical evaluation of 3D-printed zirconia crowns fabricated by selective laser melting (SLM) for posterior teeth restorations: Short-term pilot study. J. Dent. Sci..

[70] Cho S.M., Young Kim R.J., Park J.M., Chung H.M., Kim D.Y. (2024). Trueness, physical properties, and surface characteristics of additive-manufactured zirconia crown. J. Mech. Behav. Biomed. Mater..

[71] Lyu J., Wang S., Wang H., Yang X., Ma M., Liu X. (2026). Dimensional Accuracy and Adaptation of Monolithic Zirconia Crowns Fabricated by a Novel Material Jetting Technique: An In Vitro Study Based on Clinical Laboratory Data. Int. Dent. J..

[72] Su Y., Liu L., Zhang J., Deng S., Yang M., Gao S. (2025). Forming capability of advanced customized jetting additive manufacturing for restorations with different margin morphologies and surface textures. Clin. Oral Investig..

[73] Lyu J., Yang X., Li Y., Tan J., Liu X. (2023). Effect of build angle on the dimensional accuracy of monolithic zirconia crowns fabricated with the nanoparticle jetting technique. J. Prosthet. Dent..

[74] Lian Q., Wu X., He X., Meng J., Liu X., Jin Z. (2019). Accurate printing of a zirconia molar crown bridge using three-part auxiliary supports and ceramic mask projection stereolithography. Ceram. Int..

[75] Hoffmann M., Schubert N.H., Günster J., Stawarczyk B., Zocca A. (2025). Additive manufacturing of glass-ceramic dental restorations by layerwise slurry deposition (LSD-print). J. Eur. Ceram. Soc..

[76] Qi S., Sun T., Cui M., Li H., Chen Y., Zhou T., Lian Q., Shi C., Li D. (2024). Digital light processing of rare earth oxide doped natural color zirconia denture for customized aesthetic properties. Open Ceram..

[77] Kim Y.K., Han J.S., Yoon H.I. (2022). Evaluation of intaglio surface trueness, wear, and fracture resistance of zirconia crown under simulated mastication: A comparative analysis between subtractive and additive manufacturing. J. Adv. Prosthodont..

[78] Cui X., Shen Z., Wang X. (2020). Esthetic appearances of anatomic contour zirconia crowns made by additive wet deposition and subtractive dry milling: A self-controlled clinical trial. J. Prosthet. Dent..

[79] Marouki C., Shamon A., Svanborg P. (2024). Evaluation of fit and accuracy of single crowns fabricated from self-glazed zirconia compared with milled zirconia. J. Prosthet. Dent..

[80] Oh S.E., Park J.M., Kim J.H., Shim J.S., Park Y.B. (2024). Mechanical properties and crown accuracy of additively manufactured zirconia restorations. Dent. Mater..

[81] Son K., Lee J.M., Jang K.J., Lee S.K., Hwang J.H., Lee J.H., Kim H.D., Kim S.Y., Lee K.B. (2025). Effect of Pixel Offset Adjustments for XY Plane Dimensional Compensation in Digital Light Processing 3D Printing on the Surface Trueness and Fit of Zirconia Crowns. J. Funct. Biomater..

[82] Cameron A.B., Choi J.J.E., Ip A., Lyons N., Yaparathna N., Dehaghani A.E., Feih S. (2024). Assessment of the trueness of additively manufactured mol3% zirconia crowns at different printing orientations with an industrial and desktop 3D printer compared to subtractive manufacturing. J. Dent..

[83] Mou Z., Zhong J., Wang F., Alhotan A., Zhu P., Li P., Huang J. (2024). Zirconia crowns manufactured using digital light processing: Effects of build angle and layer thickness on the accuracy. J. Dent..

[84] Diken Turksayar A.A., Güven M.E., Dagistan S., Toksoy D., Önöral Ö. (2025). Comparative analysis of dimensional trueness and adaptation of 3-unit monolithic zirconia restorations fabricated with subtractive and additive technologies. J. Prosthodont..

[85] Wang W., Sun J. (2021). Dimensional accuracy and clinical adaptation of ceramic crowns fabricated with the stereolithography technique. J. Prosthet. Dent..

[86] Revilla-León M., Methani M.M., Morton D., Zandinejad A. (2020). Internal and marginal discrepancies associated with stereolithography (SLA) additively manufactured zirconia crowns. J. Prosthet. Dent..

[87] Rabel K., Nold J., Pehlke D., Shen J., Abram A., Kocjan A., Witkowski S., Kohal R.J. (2022). Zirconia fixed dental prostheses fabricated by 3D gel deposition show higher fracture strength than conventionally milled counterparts. J. Mech. Behav. Biomed. Mater..

[88] Zhao W., Singh P., Han J., Shen F., Hu K., Tsoi J.K.H. (2025). Precision-optimized process control in DLP printing of ultra-thin zirconia prostheses: A multi-factor accuracy analysis. Dent. Mater..

[89] Golriz N., Hosseinabadi N. (2024). Additive manufacturing of ceria and yttria incorporated toughened monolithic zirconia dental ceramic crowns: In vitro simulated aging behavior. J. Prosthet. Dent..

[90] Zhu H., Jiang J., Wang S., Zhou Y., Ma Y., Chen X., He F. (2025). Textured Intaglio Micropores Improve the Properties of 3D-Printed Zirconia Crowns. J. Dent. Res..

[91] Zimmermann M., Ender A., Attin T., Mehl A. (2020). Fracture load of three-unit full-contour fixed dental prostheses fabricated with subtractive and additive CAD/CAM technology. Clin. Oral Investig..

[92] Kader I.T., Özer S., Arıcan B. (2025). Evaluation of fracture resistance in 3D-printed hybrid endocrowns with different preparation designs. Turk. Endod. J..

[93] Omur Y., Toman M., Kumbuloglu O., Mutluay M., Tezvergil Mutluay A. (2026). Effect of finish line design and cementation on fracture resistance of 3D printed definitive crowns. J. Prosthet. Dent..

[94] Hatem S., Emad B., Nagi N., Mahrous A.I. (2025). Fractographic analysis of 3D printed hybrid ceramic single crowns with different aging and post-curing times: An in vitro study. Sci. Rep..

[95] Korkmaz Y.N., Turken R., Buyuk S.K., Simsek H., Yavsan A. (2026). Effects of Different Post Curing Times and Methods on Fracture Resistance and Flexural Strength of 3D-Printed Permanent Crown Resin. Int. J. Prosthodont..

[96] Suksuphan P., Krajangta N., Didron P.P., Wasanapiarnpong T., Rakmanee T. (2024). Marginal adaptation and fracture resistance of milled and 3D-printed CAD/CAM hybrid dental crown materials with various occlusal thicknesses. J. Prosthodont. Res..

[97] Turker Kader I., Ozer S., Arican B. (2025). An in vitro analysis of marginal and internal fit of 3D-printed permanent molar endocrowns with different preparation designs. Clin. Oral Investig..

[98] Xiao P., Zheng Z., Zhang Y., Zeng Y., Yan W. (2024). Accuracy and adaptation of one-piece endodontic crowns fabricated through 3D printing and milling. J. Prosthet. Dent..

[99] Abdulkareem M.A., Al-Shamma A.M.W. (2024). Marginal adaptation and fracture resistance of 3D-printed and CAD/CAM-milled definitive resin matrix ceramic crowns. Int. J. Comput. Dent..

[100] Corbani K., Hardan L., Eid R., Skienhe H., Alharbi N. (2021). Fracture Resistance of Three-unit Fixed Dental Prostheses Fabricated with Milled and 3D Printed Composite-based Materials. J. Contemp. Dent. Pract..

[101] Metin D.S., Schmidt F., Beuer F., Prause E., Ashurko I., Sarmadi B.S., Unkovskiy A. (2024). Accuracy of the intaglio surface of 3D-printed hybrid resin-ceramic crowns, veneers and table-tops: An in vitro study. J. Dent..

[102] Ghabchi B., Mavi F., Çömlekoğlu E., Saklakoğlu I.E., Uzel I. (2025). Wear behavior of CAD-CAM zirconia, ceramic, and 3D printed nano-hybrid resin crowns for the restoration of primary and permanent molars: An in vitro study. J. Prosthet. Dent..

[103] Dönmez M.B., Demirel M., Diken Türksayar A.A., Yilmaz B. (2025). Influence of printing parameters on the fabrication and fit accuracy of additively manufactured resin-based definitive three-unit fixed partial dentures. J. Dent..

[104] Arslan E., Yildirim B., Keles A. (2026). Marginal and internal fit of permanent crowns produced by different three-dimensional printing systems: A micro-computed tomography and digital three-dimensional analysis. J. Prosthodont..

[105] Sonkaya E., Zeliha Bek Kürklü G. (2025). Additively Manufactured Definitive Crown Resins on Premolar and Molar Teeth: 2-Year Results of a Prospective Clinical Study. Int. J. Prosthodont..

[106] Atasoy S., Karademir S.A. (2025). Monomer Elution of Additive- and Subtractive-Manufacturing Resins for Permanent Restorations. Int. J. Prosthodont..

[107] Arslan Acicbe B., Deniz S., Dönmez M.B., Diken Türksayar A.A., Demirel M. (2026). Wear, fracture strength, and reliability of three-unit definitive fixed dental prostheses fabricated with different vat polymerization methods. J. Dent..

[108] Orgev A., Li R., Yilmaz B., Cakmak G. (2025). Trueness, precision, and internal fit of additively and subtractively manufactured definitive resin-based crowns. J. Prosthodont..

[109] Çakmak G., Rusa A.M., Donmez M.B., Akay C., Kahveci Ç., Schimmel M., Yilmaz B. (2024). Trueness of crowns fabricated by using additively and subtractively manufactured resin-based CAD-CAM materials. J. Prosthet. Dent..

[110] Çakmak G., Agovic D., Donmez M.B., Kahveci Ç., de Paula M.S., Schimmel M., Yilmaz B. (2023). Effect of number of supports and build angle on the fabrication and internal fit accuracy of additively manufactured definitive resin-ceramic hybrid crowns. J. Dent..

[111] Dönmez M.B., Çakmak G., Kahveci Ç., Nejat A.H., Yilmaz B., Altinci P. (2025). Effect of margin thickness and cement gap on the fabrication and fit accuracy of additively manufactured crowns in definitive resins with different filler content. J. Prosthodont..

[112] Donmez M.B., Ersöz E., Çakmak G., Diken Türksayar A.A., Altinci P., Yilmaz B. (2026). Fracture strength of additively manufactured one-piece endodontic crowns in resins for definitive use: Effect of material type, margin configuration, and pulp chamber depth. J. Prosthet. Dent..

[113] Tartuk B.K., Altıntaş E., Şengül M. (2026). Fracture Resistance of CAD/CAM Lithium Disilicate and 3D-Printed Resin Crowns with Varying Occlusal Thickness: An In Vitro Study. Materials.

[114] Bazelli N., Cakmak G., Molinero-Mourelle P., Güven M.E., Yoon H.I., Yilmaz B. (2026). Dimensional accuracy and internal fit of anterior 3-unit fixed dental prostheses fabricated by additive and subtractive manufacturing using polymer-based and ceramic restorative materials. J. Prosthet. Dent..

[115] Hekimoğlu K.N., Düzgün S., Topçuoğlu H.S. (2026). Fracture resistance of endocrowns produced by 3D printing and CAD-CAM blocks: A comparative assessment. Clin. Oral Investig..

[116] Yildirim B., Arslan E. (2025). In-vitro comparison of the marginal and internal fit of 3D-printed crown restorations produced using different intraoral scanners and resin types: A Micro-CT analysis. BMC Oral Health.

[117] Abad-Coronel C., Freire Bonilla C., Vidal S., Rosero F., Encalada Abad C., Mena Córdova N., Paltán C.A., Fajardo J.I., Aliaga P. (2025). Evaluating a Novel 3D-Printed Resin for Dental Restorations: Fracture Resistance of Restorations Fabricated by Digital Press Stereolithography. Polymers.

[118] Doumit M., Beuer F., Böse M.W.H., Nicic R., Hey J., Prause E. (2024). The colour stability of 3D-printed, non-invasive restorations after 24 months in vivo—Esthetically pleasing or not?. J. Dent..

[119] Doumit M., Beuer F., Böse M.W.H., Unkovskiy A., Hey J., Prause E. (2025). Wear behavior of 3D printed, minimally invasive restorations: Clinical data after 24 months in function. J. Prosthet. Dent..

[120] Molinero-Mourelle P., Limones A., Güven M.E., Fonseca M., Peutzfeldt A., Yilmaz B., Cakmak G. (2024). Manufacturing Accuracy, Intaglio Surface Adaptation, and Survival of Additively and Subtractively Manufactured Definitive Resin Crowns After Cyclic Loading: An In Vitro Study. Int. J. Prosthodont..

[121] Abad-Coronel C., Bravo M., Tello S., Cornejo E., Paredes Y., Paltan C.A., Fajardo J.I. (2023). Fracture Resistance Comparative Analysis of Milled-Derived vs. 3D-Printed CAD/CAM Materials for Single-Unit Restorations. Polymers.

[122] Alharbi N., Alharbi A., Osman R. (2021). Stain Susceptibility of 3D-Printed Nanohybrid Composite Restorative Material and the Efficacy of Different Stain Removal Techniques: An In Vitro Study. Materials.

[123] Sabatini G.P., Dönmez M.B., Çakmak G., Demirel M., Husain N.A.H., Sesma N., Yoon H.I., Yilmaz B. (2024). Additive manufacturing trueness and internal fit of crowns in resin modified with a commercially available ceramic composite concentrate. J. Prosthet. Dent..

[124] Li Q., Liu Y., Zhao D., Yang Y., Liu Q., Zhang Y., Wu J., Dong Z. (2025). Digital light printing of zirconia/resin composite material with biomimetic graded design for dental application. Dent. Mater..

[125] Lim J.H., Shin S.H., Jung Y.E., An H., Kim J.E. (2025). Influence of heat-assisted vat photopolymerization on the physical and mechanical characteristics of dental 3D printing resins. Sci. Rep..

[126] Peskersoy C., Acar G. (2025). In Vitro Evaluation of the Mechanical Properties of Posterior Adhesive Restorations Fabricated Using Three Different Techniques. Polymers.

[127] Farag E., Sabet A., Ebeid K., El Sergany O. (2024). Build angle effect on 3D-printed dental crowns marginal fit using digital light-processing and stereo-lithography technology: An in vitro study. BMC Oral Health.

[128] Mangano F.G., Cianci D., Pranno N., Lerner H., Zarone F., Admakin O. (2024). Trueness, precision, time-efficiency and cost analysis of chairside additive and subtractive versus lab-based workflows for manufacturing single crowns: An in vitro study. J. Dent..

[129] Baldi A., Rossi T., Stura I., Comba A., Fazioni M., Rolando C., Ferrero G., Ceruti P., Scotti N. (2025). Volumetric and linear adaptation of an indirect adhesive restoration: Comparison of chairside 3D printing and milling techniques. Appl. Sci..

[130] Shin H., Kang Y.J., Kim H., Kim J.H. (2025). Effect of cement space settings on the marginal and internal fit of 3D printed definitive resin crowns. J. Prosthet. Dent..

[131] Turker Kader I., Ozer S., Arican B. (2025). Advanced 3D Insights into the Marginal and Internal Fit of Ceramic-Filled Hybrid Endocrowns With Variable Preparations. J. Esthet. Restor. Dent..

[132] Tartuk B.K., Akın Tartuk G. (2026). Impact of Thermomechanical Aging on Marginal Fit and Fracture Resistance of CAD/CAM Endocrowns Fabricated from Different Materials. Polymers.

[133] Tartuk B.K., Altıntaş E., Akgül M.C. (2026). Effect of Tooth Preparation Design on Fracture Resistance and Marginal Adaptation of Zirconia-Reinforced Lithium Silicate and 3D-Printed Overlays. Polymers.

[134] Kahraman Z., Özden S. (2026). Effect of different definitive restoration resins and postpolymerization durations on the trueness of 3D printed one-piece endodontic crowns. J. Prosthet. Dent..

[135] Güven M.E., Donmez M.B., Tezcan A.N., Yoon H.I., Yilmaz B., Çakmak G. (2024). Performance and durability of additively and subtractively manufactured resin-based onlay restorations after thermomechanical aging. J. Prosthodont..

[136] Aziz A.M., Khalifa N., Alshaibah H., Husein A.B. (2025). Influence of shoulder margin and feather-edge incisal preparations on the marginal and internal adaptation of laminate veneers fabricated with three manufacturing techniques. J. Prosthodont..

[137] Sasany R., Al-Johani H. (2026). Fabrication trueness and optical, surface, and mechanical properties of additively manufactured polymethyl methacrylate definitive crowns reinforced with silica and titanium oxide nanoparticles: An in vitro study. J. Prosthet. Dent..

[138] Altinci P., Dönmez M.B., Çakmak G., Al-Johani H., Kahveci Ç., Erkal D., Yilmaz B. (2025). Additively manufactured resin-based endocrowns: Effect of material type, margin configuration, and pulp chamber depth on fabrication trueness and fit. J. Dent..

[139] Çakmak G., Donmez M.B., Yılmaz D., Yoon H.I., Kahveci Ç., Abou-Ayash S., Yilmaz B. (2024). Fabrication trueness and marginal quality of additively manufactured resin-based definitive laminate veneers with different restoration thicknesses. J. Dent..

[140] Kakinuma H., Izumita K., Yoda N., Egusa H., Sasaki K. (2022). Comparison of the accuracy of resin-composite crowns fabricated by three-dimensional printing and milling methods. Dent. Mater. J..

[141] Orgev A., Cakmak G., Marques V., Yilmaz B., Li R. (2026). The effect of crown material type on the fracture strength of CAD-CAM fabricated crowns. J. Prosthet. Dent..

[142] Çakmak G., Donmez M.B., Molinero-Mourelle P., Kahveci Ç., Abou-Ayash S., Peutzfeldt A., Yilmaz B. (2024). Fracture resistance of additively or subtractively manufactured resin-based definitive crowns: Effect of restorative material, resin cement, and cyclic loading. Dent. Mater..

[143] Abuabboud O., Marinescu A.G., Paven M., Kovacs I.M., Nica L.M., Faur A.B., Stoia D.I., Jivănescu A. (2025). Influence of Cement Thickness, Dentine Thickness, and Intracoronal Depth on the Fracture Resistance of 3D-Printed Endocrowns: A Pilot In Vitro Study. Dent. J..

[144] Arslan Acicbe B., Deniz S., Diken Türksayar A.A., Dönmez M.B., Demirel M. (2025). Fabrication and fit accuracy of definitive resin fixed partial dentures additively manufactured using different vat polymerization technologies. J. Dent..

[145] Revilla-León M., Fry E., Supaphakorn A., Barmak A.B., Kois J.C. (2025). Manufacturing accuracy of the intaglio surface of definitive resin-ceramic crowns fabricated at different print orientations by using a stereolithography printer. J. Prosthet. Dent..

[146] Cao J., Liu X., Cameron A.B., Aarts J., Choi J.J.E. (2025). Influence of different post-polymerization methods on the dimensional accuracy of additively manufactured dental crowns. Biomater. Investig. Dent..

[147] Demirel M., Donmez M.B., Çakmak G., Dede D.Ö., Hinz S., Yilmaz B. (2025). Effect of manufacturing trinomial and restoration thickness on the fabrication trueness, fit, and margin quality of additively manufactured resin-based ultrathin laminate veneers. J. Dent..

[148] Peng P.W., Peng C.Y., Chou J.S., Chen L.X., Chao C.W., Takahashi H., Lee W.F. (2026). Effect of thickness and aging on key mechanical properties of 3D-printed crowns. Eur. J. Oral Sci..

[149] Nagai T., Alfaraj A., Chu T.G., Yang C.C., Lin W.S. (2025). Color stability of CAD-CAM hybrid ceramic materials following immersion in artificial saliva and wine. J. Prosthodont..

[150] Sasany R., Mosaddad S.A., Diaz P., Revilla-León M., Gómez-Polo M. (2025). Additively Versus Subtractively Manufactured Endocrowns: Fit, Trueness, and Retention of Zirconia, Resin Composite, and Lithium Disilicate: An In Vitro Study. Int. J. Prosthodont..

[151] Sasany R., Ucar S.M., Mosaddad S.A., Rodríguez Alonso V. (2025). Effect of polydopamine surface treatment on the mechanical properties of zirconia and resin occlusal veneers fabricated using additive and subtractive manufacturing: An in vitro study. J. Dent..

