# Supplementary material for: Three-Dimensional Printed Materials for Definitive Ceramic and Resin Restorations: A Scoping Review
Source: Materials (Basel). 2026 Sep 1;19(17):3728. doi: 10.3390/ma19173728 (PMC13567340; doi:10.3390/ma19173728)
Supplement: Supplementary file 1 [file materials-19-03728-s001.zip › Tables S1-S3. Study characteristics and extracted data.pdf]

Supplementary Table S1. Study characteristics and extracted data for additively manufactured ceramic-based restorations (n = 50).

Author (Year)	Study Design	Material	Manufacturer	Material Category	Material Composition	Printing Technology	Post-Curing Protocol	Restoration Type	Outcomes Evaluated	Main Findings
Camargo et al. (2022) [20]	In vitro comparative study	XJet printed zirconia vs laboratory and chairside milled zirconia	XJet	Zirconia ceramic	3Y-TZP zirconia	Inkjet ceramic printing	Washing, debinding, sintering	Full-contour zirconia crown	Trueness, cement-space characteristics (micro-CT), crown adaptation	Printed zirconia crowns demonstrated manufacturing accuracy intermediate between chairside and laboratory milling. Adaptation was considered clinically acceptable, with intaglio marginal accuracy strongly influencing cement-space characteristics.
Su et al. (2025) [42]	In vitro study	DLP-printed zirconia crowns	3 mol% Y_2O_3-stabilized zirconia	Zirconia ceramic	3Y-TZP zirconia	DLP	Debinding → sintering at 1400, 1450, 1500 or 1550°C (optimum 1500°C)	Printed zirconia specimens/crowns	Relative density, flexural strength, compressive strength, hardness, cytotoxicity, inflammatory response, cell adhesion	Zirconia sintered at 1500°C demonstrated the best mechanical properties together with excellent biocompatibility.
Kalman & Tribst (2024) [43]	In vitro pilot study	LithaCon 3Y 210 zirconia (printed) vs BruxZir Esthetic zirconia (milled)	Lithoz (Austria); Glidewell (USA)	Zirconia ceramic (3Y-TZP)	3 mol% yttria-stabilized zirconia (printed); monolithic BruxZir Esthetic zirconia (milled)	Lithography-based Ceramic Manufacturing (LCM; Lithoz CeraFab 7500)	Debinding followed by sintering; ~20% linear shrinkage compensated by Lithoz preprocessing software; printed at 25 µm layer thickness and 40 µm pixel size	Monolithic anterior crowns and laminate veneers	Precementation quality assessment (morphology, marginal integrity, surface quality, structural detail, esthetics, fit), clinical acceptability checklist, comparison with milled zirconia	3D-printed zirconia crowns and veneers demonstrated clinically acceptable precementation quality comparable to conventionally milled restorations.
Ganesh Kumar et al. (2025) [44]	In vitro comparative study	Zircopax zirconium silicate filament (printed) vs Upcera multilayer zirconia (milled)	Virtual Foundry; Upcera	Zirconia ceramic	Zirconium silicate filament containing zirconia (≈60 wt%) with yttria; multilayer zirconia comparator	Fused Deposition Modelling (FDM)	Debinding at 482°C for 4 h followed by sintering at 1450°C	Three-unit fixed dental prosthesis	Marginal fit, internal adaptation, trueness, precision	FDM-fabricated FDPs showed clinically acceptable marginal fit, superior precision, and comparable trueness to milled restorations. FDM was proposed as a

										feasible alternative for zirconia FDP fabrication.
Refaie et al. (2023) [45]	In vitro comparative study	Lithoz 210 3Y vs IPS e.max ZirCAD LT	Lithoz; Ivoclar	Zirconia ceramic	3Y-TZP zirconia	LCM/DLP ceramic printing (CeraFab7500)	Debinding and sintering to 1450°C; sandblasting (50 µm Al_2O_3)	Monolithic single crown	Marginal gap (VMGT), marginal gap (silicone replica), internal fit	Milled zirconia crowns demonstrated significantly smaller marginal gaps than printed crowns. Nevertheless, 3D-printed zirconia crowns exhibited clinically acceptable marginal adaptation and internal fit, supporting their clinical feasibility.
Hassan et al. (2025) [46]	In vitro comparative study	LithaCon 210 3Y	Lithoz	Zirconia ceramic	3 mol% yttria-stabilized zirconia (3Y-TZP)	Lithography-based ceramic manufacturing (LCM/DLP; CeraFab S65)	Debinding and sintering according to manufacturer	Monolithic zirconia crown	Marginal gap, internal fit (triple scan protocol), 3D deviation maps	Printed zirconia crowns exhibited significantly greater marginal and internal gaps than milled zirconia crowns; however, all measured values remained within clinically acceptable limits.
Refaie et al. (2024) [47]	In vitro comparative study	LithaCon 210 3Y; IPS e.max ZirCAD LT	Lithoz; Ivoclar	Zirconia ceramic	3 mol% Y-TZP zirconia	LCM/DLP (CeraFab7500)	Cleaning (LithaSol20), debinding, sintering at 1450°C	Monolithic zirconia crown	Trueness, precision (RMS deviations)	Milled zirconia crowns demonstrated significantly higher trueness, whereas 3D-printed crowns exhibited significantly higher precision. Both fabrication techniques produced clinically acceptable dimensional accuracy.
Zhu et al. (2025) [48]	In vitro comparative study	NPJ zirconia; VITA YZ HT; UPCERA MT	XJet; VITA Zahnfabrik; UPCERA	Zirconia ceramic	3Y-TZP zirconia	Nanoparticle Jetting (NPJ)	Water support removal (6–8 h), debinding and sintering; milled groups sintered according to manufacturers' protocols	Monolithic zirconia crown	Trueness, crown fit (triple-scan), uniformity, marginal quality	NPJ-manufactured zirconia crowns showed significantly better overall trueness and superior margin quality than milled zirconia, while crown fit remained clinically acceptable and comparable among all groups. NPJ is a promising technology for fabricating monolithic zirconia crowns.

Hassan et al. (2025) [49]	In vitro comparative study	LithaCon 210 3Y; Nacera Zirconia	Lithoz; DOCERAM Medical Ceramics	Zirconia ceramic	3 mol% yttria-stabilized zirconia (3Y-TZP)	Lithography-based Ceramic Manufacturing (LCM; CeraFab S65)	Cleaning with LithaSol 30, debinding and sintering in a single-step furnace cycle; polished, glazed and thermocycled (5,000 cycles)	Monolithic zirconia crown	Fracture resistance, Weibull analysis, surface roughness, microhardness, SEM fractography	Printed zirconia crowns showed fracture resistance comparable to milled zirconia with higher Weibull reliability. However, printed zirconia exhibited significantly greater surface roughness and lower microhardness before glazing. Glazing significantly reduced surface roughness in both groups.
Elsayed et al. (2025) [50]	In vitro comparative study	INNI-CERA BCM-W500; NexxZr.T Multi	AON ZIPRO Dental; Sagemax	Zirconia ceramic	3Y-TZP zirconia slurry; multilayer zirconia blank (3Y/5Y-TZP)	DLP	Cleaning, debinding and sintering after printing; thermomechanical aging after cementation	Monolithic zirconia crown	Marginal gap before cementation, after cementation, and after aging	Printed zirconia crowns exhibited significantly larger marginal gaps than milled zirconia before cementation, after cementation, and after thermomechanical aging. Nevertheless, aging increased marginal discrepancies in both groups.
Wang et al. (2019) [51]	In vitro comparative study	3DMIX ZrO_2 paste	3DCeram	Zirconia ceramic	Zirconia slurry with photosensitive resin binder	SLA (CERAMAKER 900)	Thermal debinding followed by sintering; no polishing or finishing	Monolithic zirconia crown	Trueness of external, intaglio, marginal and occlusal surfaces	The trueness of 3D-printed zirconia crowns was not inferior to conventionally milled CAD/CAM zirconia crowns for all evaluated surfaces, demonstrating that stereolithography can produce zirconia crowns with clinically acceptable dimensional accuracy.
Refaie et al. (2023) [52]	In vitro comparative study	LithaCon 210 3Y; IPS e.max ZirCAD LT	Lithoz; Ivoclar Vivadent	Zirconia ceramic	3Y-TZP zirconia	Lithography-based Ceramic Manufacturing (LCM)	Preconditioning, debinding, sintering; crowns cemented and subjected to 1.2 million loading cycles	Monolithic zirconia crown	Fracture resistance before and after cyclic loading, SEM fractography	Both fabrication technique and cyclic fatigue significantly influenced fracture resistance. Printed zirconia crowns showed lower fracture resistance than milled crowns before and after cyclic loading but maintained

										fracture loads well above physiological masticatory forces, indicating potential clinical applicability.
Li et al. (2023) [53]	In vitro comparative study	Zirconia suspension (47 vol%)	Porimy 3D Printing Technology	Zirconia ceramic	Resin-based zirconia suspension	SLA (CSL100)	Debinding and sintering (1500°C for milled reference; two-stage thermal treatment for printed crowns)	Monolithic zirconia crown	3D trueness, adaptation (triple-scan), RMS deviation	SLA with an occlusal full-supporting base significantly improved external trueness compared with pillar supports. Adaptation was not significantly different from milled crowns and remained clinically acceptable.
Lüchtenborg et al. (2022) [54]	In vitro comparative study	Multiple zirconia systems (SLA, DLP×2, Material Jetting, CAD/CAM)	Various manufacturers	Zirconia ceramic	3Y-TZP ceramic suspensions/inks	SLA, DLP, Material Jetting	Debinding and sintering after printing	Four-unit monolithic FDP	Manufacturing accuracy (RMSE), inner, outer and marginal deviations	Subtractive manufacturing remained the most accurate overall. Material jetting and SLA achieved accuracy comparable to milling in several regions, whereas one DLP system demonstrated the poorest accuracy. Additive manufacturing showed promise but technology-dependent variability.
Wang et al. (2025) [55]	In vitro comparative study	Zrcoop	Thales Medtech	Zirconia ceramic	3Y-TZP zirconia	Nano Particle Jetting (NPJ/Advanced Custom Jetting)	Water-soluble support removal, drying, sintering	Three-unit FDP	Trueness, intaglio fit, marginal fit, surface roughness, marginal quality	NPJ-manufactured FDPs showed significantly higher trueness, better marginal quality and fewer marginal defects than CAD/CAM milled restorations while maintaining clinically acceptable fit. Surface roughness was higher than in milled restorations.
Cai et al. (2025) [56]	Randomized controlled clinical trial (12 months)	Zrcoop	Thales Medtech	Zirconia ceramic	3Y-TZP zirconia	Nano Particle Jetting (NPJ)	Debinding, sintering, staining and veneering (0.3 mm porcelain)	Posterior single crowns	FDI criteria, PES/WES, periodontal parameters, patient satisfaction	NPJ-printed zirconia crowns demonstrated clinical performance comparable to CAD/CAM-milled crowns after 12 months. No significant differences

										were found in most clinical outcomes. Marginal adaptation was excellent (95.8% clinical success), and patient satisfaction remained high in both groups.
Lerner et al. (2021) [57]	In vitro comparative study	Lithoz Cerafab S65	Lithoz	Zirconia ceramic	Lithography-based ceramic manufacturing (LCM) zirconia	LCM (SLA-based)	Sintering according to manufacturer instructions	Monolithic zirconia crown	Trueness, marginal trueness, occlusal trueness, clinical precision (marginal adaptation and proximal contacts)	Milled crowns demonstrated significantly greater trueness than LCM-printed crowns across all evaluated regions. However, both groups exhibited high clinical precision, with comparable marginal adaptation and interproximal contacts suitable for clinical application.
Lee et al. (2024) [58]	In vitro comparative study	3DMIX ZrO_2 paste; INNI-Cera	3DCeram; AON	Zirconia ceramic	Zirconia slurry (ceramic paste with resin binder)	SLA (CERAMAKER 900) and DLP (ZIPRO)	SLA: debinding + sintering; DLP: combined debinding/sintering according to manufacturer	Monolithic zirconia crown	Trueness, precision, external, intaglio and marginal accuracy (RMS)	Milled crowns demonstrated the highest accuracy. DLP outperformed SLA, but both printed groups exhibited clinically acceptable trueness and precision suitable for clinical application.
Li et al. (2021) [59]	In vitro comparative study	47 vol% zirconia suspension	Porimy (SLA); Aidite (milling)	Zirconia ceramic	3 mol% Y-TZP zirconia suspension	SLA	Ultrasonic cleaning, two-stage debinding and sintering (1450°C/2 h for milled controls)	Monolithic zirconia crown	Fabrication accuracy (RMS), margin quality, margin chipping	SLA and milling produced comparable overall accuracy. Finish-line design significantly affected accuracy. SLA exhibited smoother margins with fewer small chippings, although knife-edge preparations resulted in large defects with both techniques.
Dagistan et al. (2025) [60]	In vitro comparative study	3DMix Zirconia 3Y; LithaCon 3Y 210; Inni Cera	3DCeram; Lithoz; AON	Zirconia ceramic	3 mol% Y-TZP	SLA, DLP and LCM	Cleaning of green body, debinding and sintering (protocol dependent on technology)	Monolithic 3-unit FDP	Fracture load, Weibull reliability, SEM fractography, EDS analysis	Subtractive manufacturing produced the highest fracture load. Among additive technologies, LCM showed the highest

										fracture resistance, followed by SLA and DLP. Nevertheless, all printed restorations exceeded clinically relevant posterior occlusal loads.
Zhai et al. (2025) [61]	In vitro comparative study	Zirconia crowns	Porimy (SLA); Lithoz (DLP); Upcera (CNC control)	Zirconia ceramic	Zirconia ceramic	SLA and DLP	Debinding and sintering according to manufacturer	Monolithic zirconia crown	Static fracture resistance, fatigue resistance, SEM fractography	CNC crowns demonstrated the highest fracture resistance, followed by DLP and SLA. Fatigue cycling did not significantly reduce fracture strength. Both SLA- and DLP-printed crowns exceeded normal clinical occlusal forces and survived all fatigue protocols, indicating satisfactory mechanical performance.
Rues et al. (2025) [62]	In vitro comparative study	3Y-TZP zirconia FDPs	AON (3D printing); Dentsply Sirona Cercon (milling)	Zirconia ceramic	3 mol% yttria-stabilized tetragonal zirconia polycrystal (3Y-TZP)	DLP	Cleaning, debinding, final sintering, artificial aging	Monolithic 3-unit FDP	Fracture resistance	Milled FDPs exhibited significantly higher fracture resistance than 3D-printed FDPs. Nevertheless, all printed restorations exceeded clinically relevant fracture thresholds, supporting their potential clinical use. Wall thickness increased fracture resistance only in milled restorations.
Kostunov et al. (2026) [63]	In vitro comparative study	3Y-TZP zirconia FDPs	AON (3D printing); Dentsply Sirona Cercon (milling)	Zirconia ceramic	3 mol% yttria-stabilized tetragonal zirconia polycrystal (3Y-TZP)	DLP	Cleaning, debinding, sintering	Monolithic 3-unit FDP	Marginal fit, internal fit	Milled FDPs demonstrated the best marginal fit. Addition of a stiffening support frame significantly improved the fit of 3D-printed FDPs, particularly thin (0.6-mm) restorations, resulting in clinically acceptable adaptation.
Li et al. (2023) [64]	In vitro comparative study	Photosensitive zirconia slurry	Porimy	Zirconia ceramic	47 vol% zirconia powder + 53 vol% photosensitive resin	SLA	Ultrasonic cleaning, debinding, sintering (1500°C)	Monolithic zirconia crown	Trueness, precision, marginal adaptation, internal adaptation	SLA demonstrated comparable trueness and marginal/internal

										adaptation to CAD/CAM milling, while precision was significantly better in the SLA group. All measured values were within clinically acceptable ranges.
Li et al. (2019) [65]	In vitro study	Resin-based zirconia suspension	Porimy	Zirconia ceramic	45 vol% zirconia suspension	SLA	Ultrasonic cleaning, debinding, sintering	Monolithic zirconia crown	Density, flexural strength, Weibull modulus, phase composition, microstructure, internal & marginal adaptation	SLA zirconia achieved a flexural strength of 812 ± 128 MPa, sufficient for crown fabrication. However, internal and marginal adaptation remained suboptimal for direct clinical application.
Toksoy & Önöral (2024) [66]	In vitro comparative study	3DMix Zirconia 3Y; LithaCon 3Y 210	3DCeram; Lithoz	Zirconia ceramic	3 mol% yttria-stabilized zirconia (3Y-TZP)	SLA, DLP, and LCM; comparator: subtractive milling	Cleaning of green body, debinding, sintering, glazing, followed by 1-year artificial aging	Three-unit monolithic zirconia FDP	Marginal, axial, axio-occlusal, and occlusal fit after sintering, glazing, and aging	LCM demonstrated the best fit, followed by SLA, subtractive manufacturing, and DLP. Glazing and artificial aging significantly affected discrepancy values, but all marginal discrepancies remained below the clinically acceptable threshold (120 µm).
Cho et al. (2025) [67]	In vitro comparative study	Multiple commercial zirconia AM systems	Various manufacturers	Zirconia ceramic	Y-TZP zirconia	Four DLP technologies and one SLA technology; comparator: subtractive milling	Debinding and sintering according to manufacturers' protocols	Monolithic zirconia crown	Marginal/internal fit, Vickers hardness, shear bond strength, SEM surface characterization	All AM crowns demonstrated clinically acceptable marginal fit (<120 µm). SLA-spreading technology produced fit and hardness comparable to or superior to subtractive manufacturing, indicating that certain AM systems are clinically promising.
Abualsaud & Alalawi (2022) [68]	In vitro comparative study	3DMix ZrO_2	3DCeram	Zirconia ceramic	3Y-TZP zirconia paste	SLA (CERAMAKER C900 Flex); comparator: CAD/CAM milling	Sintered according to manufacturer instructions	Monolithic zirconia crown	Internal fit, marginal adaptation, trueness, precision	Printed zirconia crowns showed internal fit and marginal adaptation comparable to milled crowns. Printed crowns exhibited higher overall precisionand better trueness of the occlusal

										and axial surfaces, whereas milled crowns demonstrated better trueness of the intaglio surface.
Kao et al. (2023) [69]	Prospective pilot clinical study	3D-printed zirconia crown	Taiwan Innovative Biomedical (TIB), Taiwan	Zirconia ceramic	Y-TZP zirconia	Selective Laser Melting (SLM) (as reported by the authors)	Laboratory fabrication followed by adhesive cementation with RelyX U200	Posterior single crowns	Modified USPHS/CDA criteria, plaque index, gingival index, probing depth, marginal integrity, antagonist wear, clinical complications	At 24 weeks, 100% of crowns remained clinically acceptable according to modified CDA criteria. No deterioration in probing depth or significant antagonist wear was observed. Overall, the printed zirconia crowns demonstrated favorable short-term clinical performance and periodontal compatibility.
Cho et al. (2024) [70]	In vitro comparative study	Multiple commercial zirconia AM systems	Various manufacturers	Zirconia ceramic	Y-TZP zirconia	Four DLP technologies and one SLA technology; comparator: CAD/CAM milling	Ethanol cleaning, debinding and sintering according to manufacturer protocols	Monolithic zirconia crown	Trueness, voids (micro-CT), crystal structure (XRD), surface roughness	All printed zirconia crowns demonstrated clinically acceptable trueness. However, all additive-manufactured groups contained internal voids and significantly rougher surfaces than milled zirconia.
Lyu et al. (2026) [71]	In vitro comparative study	Zrcoop zirconia (printed) vs ST zirconia (milled)	Thales Medtech; Upcera	Zirconia ceramic	Zirconia suspension (~45% zirconia particles)	Material jetting (ACJ; PGJ180); comparator: subtractive milling	Sintering at 1450 °C for 4 h (ACJ); milled crowns sintered at 1530 °C for 2 h	Monolithic zirconia crown	Trueness (RMS), marginal and internal adaptation (dual scan)	ACJ crowns demonstrated significantly higher dimensional accuracy than milled crowns in all regions, with smaller marginal and occlusal gaps in molars and smaller axial gaps in incisors. Both manufacturing methods remained within clinically acceptable ranges.
Su et al. (2025) [72]	In vitro comparative study	ACJ-printed zirconia vs ST-Color zirconia (milled)	Pugong Intelligent; Argen	Zirconia ceramic	Zirconia (composition not further specified)	Advanced customized jetting (ACJ; PG480, 10 µm layer thickness, 1200	Rinsing to remove supports, drying, debinding and sintering at 1450 °C	Crown with four margin designs (heavy-chamfer,	Tissue surface accuracy (RMS; marginal and non-marginal regions), margin morphology, surface texture accuracy (SEM, 3D profilometry)	Printed zirconia restorations showed significantly lower RMS values than milled ones in

						DPI); comparator: subtractive milling (Upcera X5)		chamfer, shoulder, BOPT)		non-marginal regions for all margin designs and in marginal regions for heavy-chamfer, chamfer and BOPT, with no difference at shoulder margins. Circular and hexagonal surface textures achieved the highest printing accuracy and pentagram the lowest.
Lyu et al. (2023) [73]	In vitro study	C800 zirconia ink	Xjet	Zirconia ceramic	~45% zirconia powder stabilized by 3 mol% yttrium oxide	Nanoparticle Jetting (NPJ; Carmel 1400C, Xjet)	Support removal by immersion in demineralized water (6–8 h), air drying overnight, debinding at 600°C for 1 h, sintering at 1450°C for 4 h	Monolithic zirconia complete crown (incisor and molar)	Dimensional accuracy (overall, external, marginal, intaglio RMS trueness), influence of build angle	Build angle significantly affected the external, marginal, and intaglio accuracy of NPJ-printed zirconia crowns. Build angles of 90° and 135° were optimal for incisors and 0° and 180° for molars. All build angles produced clinically acceptable dimensional accuracy (<100 μm).
Lian et al. (2019) [74]	In vitro study	Yttria-stabilized zirconia ceramic slurry (40 vol% solid content)	NR (bespoke ceramic MPSL machine; slurry prepared in-house)	Zirconia ceramic	Yttria-stabilized zirconia (D50 = 200 nm) in acrylic-based premix, 40 vol% solid content	Mask Projection Stereolithography (MPSL/DLP)	100 h debinding (up to 900°C), sintering at 1500°C for 1 h (air atmosphere); rescaling factors applied (1.337 X–Y, 1.401 Z)	Molar crown bridge (three-unit, single missing tooth)	Dimensional accuracy/error (green body and sintered), marginal fit, density, three-point bending strength, Weibull modulus, crystal phases (XRD), microstructure (SEM), composition (EDS)	Using three-part occlusal auxiliary supports, MPSL produced a zirconia molar crown bridge with a green-body dimensional error of ±150 μm and 99.3% relative density. Three-point bending strength was 541 ± 160 MPa — higher than human dentin but below CAD/CAM zirconia. Marginal fit and mechanical properties still required optimization, but the process was capable of individualized fabrication of complex zirconia crown bridges.
Hoffmann et al. (2025) [75]	In vitro study	Feldspar glass-ceramic slurry (custom feldspar	VITA Zahnfabrik (both feldspar	Feldspar glass-ceramic	Feldspar glass-ceramic (custom powder in water-	Layerwise Slurry Deposition (LSD-	nk cured at 120°C for 16 h, water immersion and washing, drying; two-stage debinding	Anterior single crown and front-tooth	Printing accuracy (μCT nominal-actual geometric deviation, as-printed and as-fired), esthetic appearance	LSD-printed veneers reproduced the nominal geometry well in the as-

		powder); comparator: VITABLOCS Mark II (milled)	powder and comparator blocs)		based slurry, 65.3 wt% feldspar); comparator: VITABLOCS Mark II feldspar glass-ceramic	print; slurry-based binder jetting)	(to 550°C) and firing (to 1190°C, 15 min hold) — total debinding + firing 45 min; shrinkage compensation applied to CAD models	veneer geometries (Class 1a restorations)		printed state (>95% of the surface within 100 µm deviation), but deviations up to 300 µm appeared after firing due to viscous-flow deformation, giving inadequate fit at the current stage. Esthetically high-quality restorations were achieved after individualization. LSD-print was shown capable of fabricating Class 1a feldspar glass-ceramic restorations per DIN EN ISO 6872:2019-01, though shape retention during firing needs further development.
Qi et al. (2024) [76]	In vitro study	Rare earth oxide-doped 3Y-TZP zirconia slurry (custom; doped with Pr_6O_{11}, CeO_2, Er_2O_3)	Tosoh	Zirconia ceramic	Yttria-stabilized (3Y-TZP) zirconia doped with rare earth oxides (Pr_6O_{11}, CeO_2, Er_2O_3) for natural color; 40 vol% zirconia slurry	DLP (Craftsman 150, Shaanxi Ketao-AM; 405 nm, 50 µm pixel, 31.25 µm layer thickness; multi-material for gradient coloring)	Green parts cleaned with PPG 400 resin to remove residual slurry; pyrolysis/debinding (air and argon atmospheres) and sintering per optimized temperature curves (up to ~1550°C)	Anterior single crown and incisor triple (3-unit) crown bridge	Forming/dimensional accuracy (micro-CT deviation), color accuracy (ΔE vs Vita 3D shade, NBS), color gradient reproduction (top-to-bottom)	DLP with rare earth-doped zirconia produced natural-color dentures covering the Vita 3D shade range, reproducing the top-to-bottom color gradient of natural teeth. The triple-crown bridge achieved a forming accuracy of 67.7 µm and color difference of 1.35 NBS (zone 1) and 0.97 NBS (zone 3), both below the 1.5 NBS perceptibility threshold, meeting clinical requirements.
Kim et al. (2022) [77]	In vitro comparative study	Printable zirconia suspension (AM-DLP-3YZ, M.O.P; AM-SLA-3YZ, 3D Ceram) vs KATANA STML (SM-4YZ, milled) vs KATANA UTML (SM-5YZ, milled)	M.O.P; 3D Ceram; Kuraray Noritake Dental	Zirconia ceramic	Printed groups — 3 mol% yttria-stabilized tetragonal zirconia (3Y-TZP) suspension; comparators — 4 mol% yttria partially stabilized zirconia (4Y-PSZ), 5 mol% yttria partially stabilized zirconia (5Y-PSZ)	DLP (Octave Light R1) and SLA (C100 EASY FAB, 3D Ceram); comparator: 5-axis milling (Arum 5X-300)	Layering, post-processing and sintering conducted by manufacturer (3D Ceram) or per manufacturer's instructions (M.O.P); no additional grinding or polishing performed on intaglio surfaces after sintering	Monolithic full-contour mandibular first molar single crown	Intaglio surface trueness (RMS: inner, occlusal, margin, axial), antagonist wear volume loss after simulated mastication, fracture load before and after hydrothermal/thermomechanical aging, SEM fractography	Both printed 3Y-TZP groups showed higher marginal RMS but lower occlusal and axial RMS than the milled groups, with inner-surface trueness within 50 µm and comparable across all groups. Antagonist wear showed no significant differences among

										groups. The DLP-printed group had the highest fracture load before and after aging, and only the milled groups showed a significant fracture-resistance decrease after aging. Additively manufactured 3Y-TZP crowns were clinically acceptable and comparable to or better than milled 4Y-/5Y-PSZ crowns.
Cui et al. (2020) [78]	Self-controlled clinical trial (mean 12.2-month follow-up)	Self-Glazed Zirconia (SGZ, additive wet deposition) vs Zenostar (subtractive dry milling)	Erran Tech; Wieland Dental	Zirconia ceramic	Yttria partially stabilized zirconia polycrystal (self-glazed zirconia with gradient nanostructure, printed); comparator: monolithic zirconia (milled)	Additive 3D gel deposition (wet deposition); comparator: 5-axis dry milling (Zenotec Select Hybrid)	Occlusal fissures of wet-deposition crowns and overall external surface of dry-milled crowns colored with coloring liquid (Zenostar Color Zr) before sintering and polishing	Anatomic contour monolithic premolar single crown	Color difference (ΔE) between crown and natural control tooth, influence of core/foundation type, subjective color matching (EVRSAM, VAS), crown survival, marginal fitness, marginal staining, anatomic form (modified USPHS)	Additive wet-deposition zirconia crowns showed significantly lower ΔE (better color match) and higher subjective color-matching scores (EVRSAM, VAS) than dry-milled crowns. Darker foundations (gold cast post-and-core) significantly increased crown ΔE. At mean 12.2-month follow-up, crown survival was 100% with all crowns rated Alfa for marginal fitness and staining, supporting the superior esthetics of wet-deposition crowns in color and translucency gradient.
Marouki et al. (2024) [79]	In vitro comparative study	Self-glazed zirconia (SGZ, printed); comparator: CopraSupreme milled zirconia (MZ)	ErranTech Co Ltd; comparator: Whitepeaks Dental Solutions GmbH	Zirconia ceramic	Self-glazed zirconia — zirconia slurry (ceramic particles + premixed solution of monomers, cross-linking agent, deionized water); comparator: 4 mol% yttria-stabilized zirconia (CopraSupreme)	Additive 3D gel deposition (precision 3D gel deposition, screw-extruder printing with catalyst and initiator); comparator: subtractive milling (vhf K5)	After build, intaglio surface milled, finished with surface gelation (polymerization of organic monomers), then sintered	Maxillary premolar monolithic single crown	Marginal fit (silicone replica and direct-view techniques), internal fit (chamfer, axial, occlusal), internal accuracy relative to preset cement space	Both self-glazed and milled zirconia crowns produced clinically acceptable marginal and internal fit. Self-glazed zirconia showed significantly smaller occlusal, internal, and total mean gaps than milled zirconia, and higher internal accuracy relative to the 80-μm

										preset cement space (27 μm vs 49 μm). With the direct-view technique the printed crowns also showed significantly lower marginal values, though the silicone replica technique found no significant marginal difference between the two.
Oh et al. (2024) [80]	In vitro comparative study	3DMAT500A2 zirconia paste (printed); comparator: KATANA HT milled zirconia	Genoss Co.; comparator: Kuraray Noritake Dental Inc.	Zirconia ceramic	3 mol% Y_2O_3 tetragonal zirconia (3Y-TZP); comparator: 3 mol% Y-TZP (KATANA HT)	DLP (Veltz-Cera90); comparator: subtractive milling (IDC MILL 5X, five-axis)	Post-processing (debinding and sintering) according to manufacturer's instructions (50 μm layer thickness)	Monolithic single crown (0.4 mm thickness, 40 μm cement space)	Marginal gap, internal gap (axial, line angle, occlusal) via silicone replica technique	AM-produced zirconia crowns showed significantly larger marginal and internal gaps than CNC-milled crowns at all measured sites. The printed crowns' marginal gap (82.45 μm) remained within clinically acceptable limits, and although some internal locations (axial, line angle, occlusal) exceeded the 120-μm evaluation range, all values fell within the broader 300-μm clinical acceptance range. Printed zirconia crowns were therefore less accurate than milled crowns but still within clinically acceptable limits.
Son et al. (2025) [81]	In vitro study	3DCERA zirconia	3D Controls	Zirconia ceramic	Zirconium dioxide (ZrO_2) ~94–97%, yttrium oxide (Y_2O_3) ~3–5%, aluminum oxide (Al_2O_3) <0.5%, hafnium oxide (HfO_2) <0.5%, silica trace amounts	DLP / vat photopolymerization (TD6+, 3D Controls)	Cleaning (C1 cleaner, direct injection nozzle system), debinding, and sintering (R0 furnace, up to 1600 °C); 25 μm layer thickness, 0° build orientation	Maxillary first molar single crown	Marginal and internal fit (overall, marginal, axial, occlusal gaps; triple-scan protocol, RMS), surface trueness (overall, external, intaglio; RMS, positive and negative errors), correlation between intaglio trueness and fit; influence of pixel offset (−1, 0, +1)	Pixel offset significantly affected both fit and surface trueness. Higher pixel offset increased marginal and occlusal discrepancies — a pixel offset of +1 pushed the marginal gap to 114.5 μm (approaching the 120 μm threshold), while −1 kept it clinically acceptable (85.5 μm); axial gap was unaffected. Surface

										trueness worsened with higher offset (greater positive errors), and positive intaglio errors correlated with poorer fit while negative errors improved adaptation. Optimizing pixel offset is therefore critical for clinically acceptable printed zirconia crowns.
Cameron et al. (2024) [82]	In vitro comparative study	LithaCon 3Y 230 (Lithoz, industrial) vs Inni-Cera zirconia (AON, desktop) vs IPS e.max ZirCAD Prime (milled)	Lithoz; AON; Ivoclar	Zirconia ceramic	3 mol% yttria zirconia (3Y) suspended in photopolymer (Lithoz LithaCon 3Y 230); photopolymer containing Inni-Cera zirconia powder and binding/colouring material (AON); comparator: zirconia block (IPS e.max ZirCAD Prime)	Vat polymerization with an industrial printer (CeraFab System S65, Lithoz) and a desktop printer (AON ZipPro, AON); comparator: 5-axis subtractive milling (Programat 7, Ivoclar)	Printing supports kept on samples during sintering to mitigate distortion; post-processing performed for AM groups per manufacturer instructions (specific steps not detailed); print orientations 0°, 45°, and 90°; measured layer height ~13 μm (Lithoz) and ~24.5 μm (AON)	Full-coverage mandibular first molar crown	Trueness (positive/negative average and RMS deviations) at crown margins, intaglio surface, and mesial/distal contact points; areal surface roughness (Sa) of margins; printed layer height	Printing orientation and apparatus significantly affected trueness, with the milled group performing best overall. The Lithoz 45° group was the only printed group showing no significant trueness difference from milled across all regions, while the AON group showed a clear worsening trend from 0° to 90°. Marginal areal surface roughness was within clinically acceptable limits for all groups and orientations.
Mou et al. (2024) [83]	In vitro study	ADT-ZrO_2-THP01-A	Shenzhen Adventure Tech	Zirconia ceramic	3Y-TZP; slurry of 84 wt% submicron 3Y-TZP granules (D50 ~0.6 μm) and 16 wt% photosensitive resin premix (resin monomer, photoinitiator TPO, dispersants, and other additives)	Digital light processing (DLP; ADT-3D-ZP-Printer-Pro-192-50, Shenzhen Adventure Tech), top-down, 405 nm light source	Manual support removal; cleaning with alcohol and brush; debinding in muffle furnace (minimum heating rate 0.14 °C/min, gradual heating up to 1100 °C); sintering in high-temperature muffle furnace (maximum 1500 °C, heating rate 2.5 °C/min, 2 h hold); crown scaled up 1.25× to compensate for shrinkage; build angles 0°, 45°, 90°; layer thicknesses 30 μm and 50 μm	Mandibular first molar single crown	Intaglio surface trueness, marginal trueness, precision (RMS), average and maximum deviations; influence of build angle and layer thickness	Both build angle and layer thickness significantly affected trueness and precision, with the 30 μm layer thickness offering superior accuracy over 50 μm. At 30 μm, build angles of 0° and 45° gave the best marginal trueness, while 90° gave the best intaglio trueness; the 50 μm/90° combination showed the poorest marginal and intaglio accuracy, with support-related margin defects. A 30 μm layer

										thickness with a 0° or 45° build angle was recommended, and all groups met clinical requirements.
Diken Turksayar et al. (2025) [84]	In vitro comparative study	3DMix Zirconia 3Y (SLA) vs LithaCon 210 (LCM) vs Inni Cera (DLP) vs GC Initial Zirconia HT (CNC/milled)	3D Ceram; Lithoz; Aon; GC Corporation	Zirconia ceramic	3 mol% yttria-stabilized zirconia (Y-TZP) in all groups	Stereolithography (SLA; Ceramaker C900 Flex, 3D Ceram), lithography-based ceramic manufacturing (LCM; Cerafab S65 Medical, Lithoz), and digital light processing (DLP; Zipro D, Aon); control: CNC milling (inLab MC X5, Dentsply Sirona)	SLA — rinsing in CeraClean with airbrush, two-step thermal process (debinding 600 °C/80 h, sintering 1450 °C/19 h). LCM — cleaning in LithaSol 30 with airbrush, preconditioning 120 °C/72 h, combined debinding/sintering 1450 °C/72 h. DLP — dual automated/manual cleaning with ~200 mL 83% ethyl alcohol, debinding 1100 °C/21 h, sintering 1500 °C/5 h. CNC — sintering 1500 °C. All AM groups: 150° build angle, 50 μm layer thickness	Three-unit posterior monolithic FDP (second premolar and second molar abutments, missing maxillary first molar)	Fabrication trueness (RMS) at marginal, intaglio, occlusal, and external regions; internal fit / average gap (triple-scan method)	SLA, CNC, and LCM produced similar and clinically acceptable marginal and internal trueness, while DLP showed significantly higher discrepancy in all regions, particularly marginal and intaglio. SLA had the lowest overall RMS deviation (39.88 μm) and the highest conformity, whereas DLP had the highest internal gap (218.29 μm), requiring adjustment before clinical use.
Wang & Sun (2021) [85]	In vitro study	CeraFab7500 alumina (CF) and CSL150 zirconia (CL) (printed) vs X-MILL500 zirconia (XM; milled)	Lithoz; PORIMY; XTCERA	Alumina ceramic (CF); zirconia ceramic (CL); zirconia ceramic (XM, comparator)	CF — alumina, multifunctional acrylate (solid content 49%). CL — zirconia, HDDA (1,6-hexanediol diacrylate), PET4A (pentaerythritol tetraacrylate) (solid content 49%). XM — zirconia	Stereolithography (CeraFab7500, Lithoz — LED light source, plane projection; and CSL150, PORIMY — 355 nm ultraviolet laser, point-by-point scanning); comparator: subtractive milling (X-MILL500, XTCERA)	CF and CL green bodies underwent debinding and sintering; XM underwent sintering only. Both printed systems used 25 μm layer thickness. STL oversized per each manufacturer's shrinkage ratio (CF XY:1.245/Z:1.275; CL X:1.189/Y:1.205/Z:1.294)	Maxillary right first molar single crown	Dimensional accuracy (mean absolute deviation vs reference, best-fit superimposition); clinical adaptation (marginal, axial, corner, and occlusal gaps via silicone replica method)	CeraFab7500 showed significantly better dimensional accuracy (41 μm) than CSL150 (65 μm) and the milled X-MILL500 (72 μm), with no significant difference between CSL150 and X-MILL500. X-MILL500 gave better marginal, corner, and occlusal adaptation but inferior axial adaptation compared with the printed crowns. Both SLA systems produced crowns with dimensional accuracy and marginal adaptation within clinically acceptable limits.
Revilla-León et al. (2020) [86]	In vitro comparative study	3DMix ZrO_2 paste	3DCeram Co	Zirconia ceramic	Zirconia stabilized with 3 mol% yttria	SLA (CERAMAKER 900); comparator: 5-axis milling (CARES	After printing, thermal binder removal (debinding) and sintering; no additional	Anatomically contoured monolithic zirconia crown (also	Marginal discrepancy, internal discrepancy (silicone replica	Milled zirconia crowns showed significantly lower marginal and

						zirconium-dioxide, Straumann)	finishing or polishing performed	splinted/enamel-dentin-divided AM crown design)	technique), correlation between marginal and internal discrepancies	internal discrepancies than printed crowns. Anatomically contoured printed crowns had the highest, clinically unacceptable marginal discrepancies, whereas the splinted printed design achieved clinically acceptable marginal and internal adaptation. A weak but significant correlation was found between marginal and internal discrepancies in all groups.
Rabel et al. (2022) [87]	In vitro comparative study	Self-glazed zirconia (Y-TZP_SG, printed); comparator: inCoris TZI (Y-TZP_C, milled)	ErranTech Co. Ltd.; comparator: Dentsply Sirona	Zirconia ceramic	3 mol% yttria-stabilized tetragonal zirconia (3Y-TZP; Y_2O_3 4.5–6.6%, HfO_2 ≤5%, Al_2O_3 ≤0.5%); comparator: 2nd-generation 3Y-TZP (ZrO_2+HfO_2+Y_2O_3 ≥99%, Y_2O_3 >4.5–≤6.0%)	Additive 3D ceramic gel deposition; comparator: 5-axis milling (inLab MC X5)	Self-glazed: hybrid gel deposited on dies to form green body, intaglio surface milled to refine internal surface/margin, sintering to full density (1450°C, 2 h); no surface post-processing/glazing. Comparator: milled, sintered (1510°C, 120 min), glazed twice (Ivocolor FL Glaze, 740°C)	Three-unit fixed dental prosthesis (posterior, replacing molar #16)	Fracture strength (before and after dynamic loading/aging), survival after 5 million thermocyclic loading cycles, surface layer topography/microstructure/elemental composition, bulk microstructure and phase composition, fractographic analysis	Self-glazed printed zirconia FDPs demonstrated roughly twice the fracture strength of milled and glazed FDPs, attributed to a finer-grained, more homogeneous surface microstructure with fewer flaws compared with the flaw-containing glaze layer of milled restorations. All FDPs survived 5 million loading cycles; fracture strength of the printed group decreased after aging (5165→4507 N) but remained significantly above the milled group (1923→2041 N). Fracture origins were located in surface-layer microstructural flaws in both groups.
Zhao et al. (2025) [88]	In vitro study	80 wt% 3Y zirconia slurry; comparator: IPS e.max CAD (milled)	Hangzhou Errantech; comparator: Ivoclar Vivadent	Zirconia ceramic	80 wt% 3 mol% yttria-stabilized (3Y) zirconia slurry; comparator: lithium disilicate glass-ceramic	DLP (custom top-down, 405 nm LED, 70 μm X/Y resolution, 25 μm layer thickness); comparator: 5-axis	Green bodies ultrasonically cleaned in 50:50 HDDA/ethanol (10 min), debinding (600°C, 2 h, argon), sintering (1510°C, 2 h, air)	Ultra-thin laminate veneer (0.1 mm wall thickness, 0.03 mm margin)	Intaglio surface accuracy (RMS deviation), margin integrity; influence of optimized process control (light-curing fidelity, support/overhang angle, asymmetric sintering shrinka	With an integrated process-control framework, 0.1-mm ultra-thin printed zirconia veneers achieved an intaglio accuracy of 48 ± 9

						milling (ARUM 5X-400)				μm RMS, statistically equivalent to 0.5-mm milled lithium disilicate controls (47 ± 8 μm). A critical overhang angle (~65°) minimized unsupported deformation, and asymmetric inner/outer sintering shrinkage was identified and compensated. The measured marginal width (0.08 mm) exceeded the designed 0.03 mm due to lateral light scattering, but veneers showed no visible margin gaps and clinically acceptable fit.
Golriz & Hosseinabadi (2024) [89]	In vitro study	Custom zirconia slurries — Y-TZP, Ce-TZP, and CeY-TZP formulations	Prepared in-house from Sigma-Aldrich/Merck precursors (printed on 3Dceram C900 flex)	Zirconia ceramic	Three compositions — yttria-stabilized (Y-TZP, 3 mol% yttria); ceria-stabilized (Ce-TZP, 10 mol% ceria); ceria:yttria co-stabilized (CeY-TZP, 7 mol% ceria + 3 mol% yttria); ~45 vol% zirconia slurries with HDDA wetting agent, photo-initiators, and light absorber	Combined SLA and DLP (laser-polymerized; 3Dceram C900 flex, 400 nm, 20 μm layers); comparator: none	Debinding (550°C, 2 h; 5°C/min heating, 1°C/min cooling), cold isostatic pressing (350 MPa, 2 h), pressureless sintering (1200°C or 1300°C, 2 h, air); ground (600/P1200), polished with diamond paste and 0.5-μm ultrafine polisher	Monolithic posterior crown (7 mm mesiodistal, 9 mm buccolingual, 8 mm length, 2 mm cusp/fissure thickness)	Hardness, fracture toughness (K_IC), flexural strength, modulus of elasticity, Weibull modulus, fatigue limit, translucency and contrast ratio, phase composition, density/porosity; durability after hydrothermal aging, thermocycling, and mastication simulation	All three additively manufactured zirconia compositions achieved near-theoretical density (up to 99.1%), high hardness (14–20 GPa), toughness (6–7 MPa·m^1/2), and flexural strength (~1200–1300 MPa) meeting crown requirements. The co-stabilized CeY-TZP showed the best durability, with the smallest strength reductions after hydrothermal aging (~2.5%) and thermocycling, and highest resistance to low-temperature degradation, attributed to combined ceria/yttria transformation toughening. Mastication simulation caused the largest strength drops, but co-stabilization

										minimized aging deterioration overall, supporting these materials for durable posterior crowns.
Zhu et al. (2025) [90]	In vitro study	UPCERA MT zirconia (NPJ intaglio porous crowns and standard NPJ crowns) vs UPCERA MT (milled)	UPCERA; NPJ printer: XJET (Carmel 1400); milling: AIDITE (AMD-500)	Zirconia ceramic	3 mol% (5.2 wt%) yttria-stabilized tetragonal zirconia polycrystal (3Y-TZP)	Nanoparticle Jetting (NPJ); comparator: subtractive milling	Crowns polished with ceramic ("CD" series) and gray coarse then fine yellow rubber polishers ("RD" series) using a low-speed handpiece; ultrasonic alcohol cleaning (5 min) before cementation	Monolithic mandibular first molar single crown (NPJ intaglio porous design), occlusal thicknesses 0.6, 0.8, 1.0, 1.2 mm	Surface roughness, fracture load, 2-body wear, fatigue resistance, shear bond strength (SBS), crystalline phase (XRD), grain size/microstructure (SEM), fracture mode	NPJ intaglio porous crowns showed fracture load comparable to standard NPJ crowns, though milled crowns had higher fracture load at 1.0 and 1.2 mm occlusal thickness. After polishing, printed crowns achieved occlusal roughness, 2-body wear, and aging resistance similar to milled crowns, while the intaglio porous design provided significantly superior intaglio roughness and bond strength compared with air-abraded milled zirconia. NPJ intaglio porous crowns were supported as a viable restorative option with reliable mechanical performance and enhanced bonding.

Supplementary Table S2. Study characteristics and extracted data for additively manufactured resin-based restorations (n = 57).

Author (Year)	Study Design	Material/Product	Manufacturer	Material Category	Material Composition	Printing Technology	Post-Curing Protocol	Restoration Type	Outcomes Evaluated	Main Findings
Zimmermann et al. (2020) [91]	In vitro comparative study	els 3D resin even stronger (printed) vs BRILLIANT Crios; Telio CAD; e.max CAD; inCoris TZI C (milled/ground comparators)	Saremco Dental AG (els 3D); Coltène AG; Ivoclar Vivadent AG; Dentsply Sirona	Definitive composite resin (printed); comparators: composite resin, PMMA, lithium disilicate glass-ceramic, zirconia	els 3D: methacrylate monomer organic matrix with dental glass silica inorganic fillers; comparators: BC — cross-linked methacrylates + barium glass/silicon dioxide fillers; TC — PMMA; EX — lithium disilicate (Li_2SiO_5 70%); TZ — ZrO_2+HfO_2+Y_2O_3 oxide ceramic	DLP (Asiga Freeform PRO2); comparators: subtractive milling/grinding (MCX5, inLab MCXL)	Cleaned/washed in isopropanol 2 × 5 min in ultrasonic cleaner; light-cured with 4000 exposures under nitrogen atmosphere (Otoflash G171); resin-based specimens not polished after fabrication	Three-unit full-contour fixed dental prosthesis (replacing one molar)	Fracture load after thermomechanical loading (chewing simulator, 1.2 million cycles); survival of thermomechanical loading; failure/fracture pattern	All FDPs survived thermomechanical loading. The printed composite (els 3D) showed the lowest mean fracture load (928.9 ± 193.8 N), significantly lower than the milled composite BRILLIANT Crios (1494.8 ± 214.5 N); zirconia (TZ) showed the highest (2099.5 ± 382.1 N). All groups exceeded reported posterior masticatory forces (~600 N). Particle-filled composite CAD/CAM materials achieved fracture loads within the range of ceramic materials indicated for three-unit FDPs.
Türker Kader et al. (2025) [92]	In vitro study	VarseoSmile Triniq	BEGO GmbH (Germany)	Ceramic-filled hybrid resin	Ceramic-filled hybrid permanent restorative resin	DLP	Cleaning in 99% isopropanol (5 min) → post-cure twice for 20 min at 60°C	Ceramic-filled hybrid endocrowns	Fracture resistance, failure mode	Shoulder-margin preparations showed significantly higher fracture resistance than butt-joint preparations. The study supports the use of 3D-printed ceramic-filled hybrid endocrowns, with preparation design playing a critical role in mechanical performance.
Omur et al. (2026) [93]	In vitro	VarseoSmile Crown Plus	BEGO	Ceramic-filled permanent resin	Ceramic-filled photopolymer resin	DLP (Varseo XS)	Cleaned in 96% ethanol (3 + 2 min ultrasonic), post-cured with Otoflash G171 under nitrogen (1500	Definitive premolar crown	Fracture resistance, initial failure load, acoustic emission analysis, failure mode (SEM), effect of finish line, cement type and aging	Finish line design had no significant effect on fracture resistance. Ceramic-filled printed crowns demonstrated adequate mechanical performance for definitive restorations.

							flashes/side), airborne-particle abrasion (50 µm Al_2O_3), polishing			
Hatem et al. (2025) [94]	In vitro	Saremco Print CROWNTEC	Saremco Dental	Hybrid ceramic resin	Hybrid ceramic resin (high ceramic filler content)	DLP (Asiga MAX UV)	UV post-curing using SprintRay Procure for 10 or 20 min	Single premolar crown	Fracture resistance, thermocycling aging (5,000 vs 10,000 cycles), SEM fractographic analysis, failure mode	Longer post-curing and aging showed a trend toward lower fracture resistance but without statistical significance.
Korkmaz et al. (2026) [95]	In vitro	Saremco Crowntec	Saremco Dental	Hybrid ceramic resin	Permanent crown hybrid ceramic resin	DLP (NextDent 5100)	LC-3DPrint Box; dry or glycerin immersion for 15, 30 or 45 min (405 nm UV)	Single crown	Fracture resistance, flexural strength, influence of post-curing time and medium	Increasing post-curing time improved fracture resistance and flexural strength. Thirty minutes appeared sufficient, with no additional benefit beyond 30 min. Dry and glycerin post-curing produced similar fracture resistance, although glycerin influenced flexural strength at shorter curing times.
Suksuphan et al. (2024) [96]	In vitro comparative study	VarseoSmile Crown Plus	BEGO	Hybrid ceramic/composite	Ceramic-filled hybrid composite resin	DLP (ASIGA Freeform Pro 2)	UV post-curing according to manufacturer	Single posterior crown	Marginal adaptation (micro-CT), absolute marginal discrepancy, marginal gap, fracture resistance	Printed crowns exhibited significantly better marginal adaptation than the milled hybrid materials. However, they demonstrated lower fracture resistance, although all restorations withstood physiological posterior occlusal forces even at reduced occlusal thicknesses.
Kader et al. (2025) [97]	In vitro	VarseoSmile TriniQ	BEGO	Ceramic-filled hybrid resin	Ceramic-filled permanent hybrid resin	DLP (Asiga Ultra 50)	Washed in 99% IPA (5 min), post-cured twice for 20 min at 60°C	Permanent molar endocrown	Marginal fit, internal fit, pulp chamber gap, overall adaptation	Shoulder preparation with a 2-mm pulp chamber depth demonstrated significantly better marginal and pulp chamber adaptation than the other preparation designs. Internal and overall gaps were not significantly affected by preparation design.
Xiao et al. (2024) [98]	In vitro comparative study	3DMix ZrO_2 paste; UltraPrint Dental Denture Teeth UV A2	3DCeram; HeyGears	Zirconia ceramic and resin composite	3 mol% Y-TZP zirconia slurry; permanent resin composite	SLA (zirconia); DLP (resin)	Zirconia: degreasing, drying, sintering (1500°C); Resin: IPA cleaning and post-curing (200 W, 300 s)	One-piece endodontic crown (endocrown)	Trueness, precision, marginal adaptation, internal adaptation (micro-CT)	Milled resin endocrowns showed significantly higher trueness and adaptation than printed resin restorations. For zirconia endocrowns, no significant differences were observed between printed

										and milled groups. Both printed zirconia and resin restorations demonstrated clinically acceptable adaptation.
Abdulkareem & Al-Shamma (2024) [99]	In vitro comparative study	VarseoSmile Crown Plus	BEGO	Resin matrix ceramic (ceramic-filled hybrid composite)	Ceramic-filled hybrid composite resin	DLP (Asiga Max UV)	96% ethanol ultrasonic cleaning (5 min), post-cured twice for 20 min at 60°C, polishing	Definitive full-coverage crown	Marginal adaptation (before/after cementation), fracture resistance, failure mode	The 3D-printed crowns demonstrated significantly smaller marginal gaps than both CAD/CAM-milled Vita Enamic and Cerasmart crowns before and after cementation. Fracture resistance was comparable to Cerasmart and significantly higher than Vita Enamic.
Corbani et al. (2021) [100]	In vitro comparative study	Irix Max	DWS (Italy)	Nanocomposite resin	Permanent nanocomposite resin	SLA (DFAB, DWS)	Cleaned with 95% ethanol for 1 min, UV post-cured (Dcure, DWS) for 6 min	Three-unit fixed dental prosthesis (FPD)	Fracture resistance, fatigue behavior (thermocycling and cyclic loading), failure mode	After thermocycling (5,000 cycles) and mechanical fatigue (1,200,000 cycles), no restorations exhibited cracks or chipping. The 3D-printed composite FDPs demonstrated fracture resistance (1360.2 ± 148.2 N) comparable to high-density polymer (HDP) FDPs and significantly higher than fiber-reinforced composite (FRC) FDPs, although lower than metal-ceramic controls. Most failures in the 3D-printed group were repairable, supporting the use of permanent 3D-printed composite materials for definitive three-unit FDPs.
Metin et al. (2024) [101]	In vitro study	VarseoSmile Crownplus A3	BEGO (Germany)	Ceramic-filled hybrid resin	Ceramic-filled hybrid composite resin	DLP (Varseo XS)	Ultrasonic cleaning in 96% ethanol (3 min + 2 min), sandblasting (50 µm Perlablast, 1–1.5 bar), post-cured in Otoflash (2 × 1500 flashes)	Single crown, veneer, and occlusal table-top restorations	Trueness, precision, influence of build angle on intaglio accuracy (RMS deviation)	Build angle significantly affected restoration accuracy. Crowns exhibited the highest trueness at 30°–45°, veneers at 30°, and table-tops at 0°–30°, whereas 90°produced the poorest trueness for all restorations. Appropriate build orientation improves the accuracy of definitive DLP-printed hybrid restorations.

Ghabchi et al. (2025) [102]	In vitro comparative study	VarseoSmile Crownplus	BEGO GmbH	Nano-hybrid resin	Nano-hybrid resin composite	DLP (Varseo XS)	Isopropyl alcohol cleaning followed by Otoflash light polymerization (2 × 1500 flashes)	Single crown	Wear of restorative material, antagonist wear (primary and permanent teeth), volumetric wear	The 3D-printed nano-hybrid resin produced significantly less wear of opposing primary and permanent teeth than zirconia and lithium disilicate, although the resin itself exhibited greater wear. Its wear behavior was considered more predictable and suitable for interim or minimally abrasive definitive restorations.
Dönmez et al. (2025) [103]	In vitro comparative study	VarseoSmile Triniq	BEGO GmbH	Ceramic-filled hybrid resin	Ceramic-filled hybrid composite resin	DLP (Asiga Max UV)	Ultrasonic cleaning in 96% ethanol (3 + 2 min), drying, post-cured under nitrogen (Otoflash G171, 4000 flashes)	Three-unit fixed partial denture (FPD)	Fabrication accuracy (trueness, precision), fit accuracy (average gap), influence of build orientation and layer thickness	Build orientation and layer thickness significantly affected fabrication and fit accuracy. 30° and 90° orientation with 50-µm layersyielded the highest accuracy among printed FPDs. Printed FPDs showed fabrication accuracy comparable to milled composite FPDs, although milled restorations demonstrated superior internal fit.
Arslan et al. (2026) [104]	In vitro comparative study	Saremco Crowntec; SprintRay Crown; Power Resin C&B	Saremco Dental; SprintRay; Denta Fab	Permanent composite resin	UDMA-based composite; methacrylate-based resin; Bis-GMA/TEGDMA-based composite	DLP (Asiga MAX UV, SprintRay Pro95S, DentaFab Sage)	Manufacturer-specific protocols: alcohol/special solution washing followed by UV post-curing (10–15 min depending on system)	Full-coverage crown	Marginal fit, internal fit (micro-CT and 3D superimposition)	Significant differences in fit were observed among the three printer–resin systems. SprintRay Crown demonstrated the best marginal adaptation, whereas Saremco Crowntec and Power Resin C&B showed comparable results. Both micro-CT and digital 3D analyses produced consistent findings.
Sonkaya et al. (2025) [105]	Prospective clinical study (24-month follow-up)	Permanent Crown Resin	Formlabs Dental	Ceramic-filled hybrid resin	Hybrid ceramic-filled methacrylate photopolymer containing 30–50 wt% silanized dental glass	SLA (Form 3B)	Washed in 99% isopropyl alcohol (Form Wash), post-cured twice at 60°C for 20 min (Form Cure), polished	Overlay and endocrown	Clinical performance (modified USPHS), retention, marginal adaptation, marginal discoloration, surface texture, color match, patient satisfaction	At 24 months, all restorations remained clinically acceptable (≥99.5% Alpha + Bravo scores). No failures or loss of retention occurred. Only marginal discoloration increased significantly over time, while all other USPHS

										criteria remained stable, supporting favorable short-term clinical performance of 3D-printed permanent ceramic-filled resin restorations.
Atasoy & Karademir (2025) [106]	In vitro comparative study	Crowntec; VarseoSmile Crown Plus; Cerasmart	Saremco; BEGO; GC	Hybrid resin / resin nanoceramic	Permanent printable composite resins and CAD/CAM resin nanoceramic	DLP	Manufacturer-specific cleaning, post-curing (2 × 5 min UV), polishing	Permanent resin restoration	Residual monomer elution (HPLC)	Printable permanent crown resins released significantly higher residual monomers than the CAD/CAM resin nanoceramic, although all concentrations remained below reported cytotoxic thresholds.
Arslan Acicbe et al. (2026) [107]	In vitro comparative study	VarseoSmile Triniq	BEGO (Germany)	Ceramic-filled hybrid resin	Definitive ceramic-filled resin	DLP, Low Force Display (LFD), LCD (vat photopolymerization); comparator: subtractively manufactured HIPC	Manufacturer-specific washing and post-curing according to each printing system	Three-unit fixed dental prosthesis (FDP)	Wear (maximum wear depth, volume loss), fracture strength, Weibull reliability after thermomechanical aging	Subtractive HIPC FDPs showed the greatest fracture strength and wear resistance. Among the printed groups, DLP exhibited the lowest wear (volume loss), whereas LFD demonstrated significantly higher fracture strength and characteristic strength than LCD. All AM FDPs exceeded clinically relevant posterior masticatory forces, indicating potential suitability for definitive posterior FDPs.
Orgev et al. (2025) [108]	In vitro comparative study	Pro Resins Crown X; Flexcera Smile Ultra+; Shofu HC; Brilliant Crios	Straumann; Desktop Health; SHOFU; Coltene	Definitive resin composites	Acrylic resin (Crown X), methacrylate-based resin (Flexcera), nanoparticle-filled resin composites	DLP (Straumann CARES P30); comparator: CAD/CAM milling	Printed crowns post-cured for 20 min; supports removed without additional polishing	Single definitive crown	Trueness, precision, internal fit	Manufacturing technique significantly affected fabrication accuracy. Both additive and subtractive workflows produced clinically acceptable restorations.
Çakmak et al. (2024) [109]	In vitro comparative study	Crowntec; Brilliant Crios; breCAM.monoCOM; G-CAM	Saremco; Coltene; bredent; Graphenano	Resin-based CAD/CAM materials	Printed composite resin; reinforced composite resin; PMMA; nanographene-reinforced PMMA	DLP (ASIGA MAX UV); comparator: CAD/CAM milling	Otoflash xenon curing (4000 flashes under nitrogen atmosphere)	Single definitive crown	Overall, external, occlusal, intaglio occlusal and marginal trueness	Material significantly influenced trueness.
Çakmak et al. (2023) [110]	In vitro experimental study	Crowntec	Saremco Dental AG	Resin-ceramic hybrid	Resin-ceramic hybrid composite	DLP (ASIGA MAX UV)	Otoflash G171 xenon polymerization (4000 flashes	Single definitive crown	Fabrication trueness, precision, internal fit according to build angle and support number	Build angle and support configuration influenced fabrication accuracy and precision, whereas average internal fit remained similar

							under nitrogen atmosphere)			among groups. A 30° build angle with fewer supports yielded the highest precision while preserving clinical fit.
Dönmez et al. (2025) [111]	In vitro comparative study	FREEPRINT Crown; Rodin Sculpture; Rodin Sculpture 2.0; Enamic	DETAX; Pac-Dent; VITA	Definitive hybrid resin composites and polymer-infiltrated ceramic	Ceramic-filled hybrid resins vs polymer-infiltrated ceramic network	DLP (ASIGA MAX UV); comparator: CAD/CAM milling	FREEPRINT: 4000 xenon flashes; Rodin materials: 5000 xenon flashes under nitrogen atmosphere (Otoflash G171)	Single definitive crown	Overall, external, intaglio and marginal trueness, precision, internal fit (average gap); influence of margin thickness and cement gap	Material, margin thickness, and cement gap significantly affected fabrication accuracy and fit. Higher filler-content resins generally showed lower fabrication accuracy.
Dönmez et al. (2026) [112]	In vitro comparative study	Tera Harz TC-80DP; FREEPRINT Crown; Crowntec; Tetric CAD	Graphy; DETAX; Saremco; Ivoclar	Definitive resin composites	Urethane acrylate resin; ceramic-filled hybrid resins; glass-reinforced composite	DLP (ASIGA MAX UV); comparator: CAD/CAM milling	Xenon flash polymerization (Otoflash G171, 4000 flashes under nitrogen atmosphere)	One-piece endodontic crown	Fracture strength after cyclic loading; effects of material, margin design and pulp chamber depth	All printed materials survived 1.2 million loading cyclesand demonstrated fracture loads exceeding reported posterior masticatory forces, indicating their suitability for definitive one-piece endodontic crowns.
Tartuk et al. (2026) [113]	In vitro comparative study	Saremco Dental; Ivoclar Vivadent	Saremco Dental; Ivoclar Vivadent	Definitive composite resin	BisEMA-based resin; comparator: lithium disilicate glass ceramic	SLA (Form 3, 50 µm layer thickness); comparator: subtractive milling (DWX-52D)	Rinsed in 95% isopropyl alcohol for 3 min (Form Wash), support removal, post-cured at 60 °C for 20 min (Form Cure), airborne-particle abrasion (50 µm Al_2O_3, 2 bar, 10 s)	Full-coverage crown with varying occlusal thickness (0.5, 1.0, 1.5 mm)	Fracture resistance after thermomechanical aging, failure mode (SEM)	Lithium disilicate outperformed printed resin at all thicknesses, with fracture resistance increasing with occlusal thickness in both materials. Printed crowns ≥1.0 mm exceeded masticatory forces with mostly repairable failures, whereas 0.5 mm crowns were clinically insufficient.
Bazelli et al. (2026) [114]	In vitro comparative study	Tera Harz TC-80DP (printed) vs breCAM.HIPC; G-CAM; IPS e.max CAD (milled)	Graphy; bredent; Graphenano Dental; Ivoclar	Definitive resin composite	Urethane acrylate-based polymer; comparators: high-impact polymer composite, nanographene-reinforced PMMA, lithium disilicate glass-ceramic	DLP (Asiga MAX UV, 50 µm layer thickness); comparator: subtractive milling (PrograMill PM7)	Ultrasonic cleaning in 95% ethanol (45 s), drying 30 min, post-polymerized under nitrogen (Otoflash G171, 2 × 2000 flashes per surface); no polishing or glazing	Anterior 3-unit tooth-supported fixed dental prosthesis	Trueness and precision (RMS; external, internal, marginal regions), internal fit (average gap distance, triple scan)	Printed FDPs showed the highest trueness and precision, particularly internally and marginally, but the largest internal gap. All materials achieved internal fit close to or below clinically acceptable thresholds.

Hekimoğlu et al. (2026) [115]	In vitro comparative study	Crowntec (printed) vs G Ceram; Cerasmart; Tessera (milled)	Saremco; ATLAS-ENTA; GC; Dentsply Sirona	Ceramic-reinforced composite resin	Bis-EMA with dental glass and silica fillers; comparators: leucite-reinforced glass ceramic, resin nanoceramic, lithium disilicate glass ceramic	DLP (Asiga Max, 50 µm layer thickness); comparator: subtractive milling (CEREC MCX)	Alcohol cleaning and post-curing per manufacturer; airborne-particle abrasion (110 µm Al_2O_3) and silanization	Molar endocrown with and without 1-mm ferrule	Fracture resistance, failure mode, influence of ferrule presence	Printed resin, resin nanoceramic and lithium disilicate endocrowns showed significantly higher fracture resistance than leucite-reinforced ceramic, and all groups exceeded reported maximum masticatory forces. Ferrule presence had no significant effect, and printed resin restorations showed predominantly restorable failures.
Yıldırım & Arslan (2025) [116]	In vitro comparative study	Saremco CrownTec; VarseoSmile Crown Plus	Saremco Dental; BEGO	Definitive resin composites	Bis-GMA/Bis-EMA derivatives with ceramic fillers	DLP (Asiga Max UV, 50 µm layer thickness, 0° support angle)	96% isopropyl alcohol cleaning and UV curing per manufacturer	Full-coverage crown (maxillary first premolar)	Marginal and internal fit (micro-CT; axial, coronal and sagittal sections), influence of intraoral scanner and resin type	Both scanner and resin type significantly affected marginal and internal fit in several regions, with the Primescan–VarseoSmile combination showing the lowest and the Trios–Saremco combination the highest gaps. Some marginal regions exceeded the 200 µm threshold.
Abad-Coronel et al. (2025) [117]	In vitro comparative study	Ceramic Crown (printed) vs Empress CAD; Vita Enamic; Cerasmart; Zirconia ZIRCOSTAR (milled)	SprintRay; Ivoclar Vivadent; Vita Zahnfabrik; GC; Kerox	Ceramic-filled composite resin	Methacrylate and acrylic monomers with inorganic fillers >50%; comparators: lithium disilicate, polymer-infiltrated ceramic, resin nanoceramic, zirconia	Digital press stereolithography (DPS; Midas, capsule-based resin, 385 nm); comparator: subtractive milling (PrimeMill; inLab MC X5)	99% isopropyl alcohol cleaning, automated post-curing (NanoCure), extraoral polishing	Full-coverage molar crown	Marginal, cervical, axial and occlusal gaps after thermocycling (sectioning and stereomicroscopy)	All groups showed gap values within clinically acceptable thresholds, with zirconia demonstrating the lowest gaps and variability. The printed DPS crowns showed greater variability, particularly in the occlusal and cervical regions, although mean marginal gap remained clinically acceptable.
Doumit et al. (2024) [118]	Prospective clinical study (24-month follow-up)	VarseoSmile Crown plus	BEGO	Ceramic-filled hybrid resin	Ceramic-filled hybrid material (30–50 wt% inorganic fillers; 0.7 µm silanized dental glass, methyl benzoylformate, diphenyl (2,4,6-trimethylbenzoyl) phosphine oxide)	DLP (Varseo XS)	Post-processed with 1,500 flashes twice (Otoflash); for insertion: air-abraded 50 µm Al_2O_3 (10 s), conditioned with Monobond Plus	Non-invasive single restorations (anterior labial + posterior occlusal/table-top	Colour stability (ΔE via CIELAB spectrophotometry) at 6, 12, and 24 months; colour change by tooth region	3D-printed non-invasive restorations showed steady, progressive discolouration over 24 months. After two years only 18% were rated alpha/bravo (no/hardly visible change) and 54% exceeded ΔE > 6.8 (clinically unacceptable). Premolar restorations showed the best

							primer, polished (Sof-Lex XT)			colour stability and molars the worst. Despite manufacturer recommendation for permanent use, the authors recommend only a ~6-month wearing time.
Doumit et al. (2025) [119]	Proof-of-concept prospective clinical study (24-month follow-up)	VarseoSmile Crown plus	BEGO	Definitive composite resin	Ceramic-filled hybrid material	NR (Varseo XS printer; 50 µm layer thickness; build angle 35–45°); comparator: none	Ultrasonic cleaning in 99% isopropanol (3 min rough + 2 min final), dried in oil-free airflow; airborne-particle abrasion with glass beads (Perlablast micro, 0.15 MPa); light-polymerized with Otoflash (2 × 1500 flashes, 2 xenon lamps, 10 Hz, 300–700 nm); glazed (Optiglaze Clear HV)	Noninvasive occlusal veneers and table-tops (posterior); facial and incisal veneers (anterior)	Maximum occlusal height loss, mean profile loss (intraoral scanning + 3D metrology superimposition) at 12 and 24 months; fracture classification; survival rate	Molar restorations showed the highest maximum occlusal height loss (1.25 mm at 24 months); anterior restorations the lowest. 35% of restorations (123/352) had wear >0.5 mm and were classified as fractured at 24 months. The material showed considerable wear and appears suitable only for short-term interim use.
Molinero-Mourelle et al. (2024) [120]	In vitro comparative study	VarseoSmile Crown Plus (AM-HRC); Crowntec (AM-RC); Vita Enamic (SM-PICN, milled comparator)	Bego; Saremco Dental; VITA Zahnfabrik	Definitive printed hybrid resin composite and glass filler-reinforced resin composite; comparator: milled polymer-infiltrated ceramic network	No detailed chemical composition	DLP (Asiga MAX UV); comparator: subtractive milling (PrograMill PM7)	AM-HRC: ultrasonic cleaning in 95% ethanol (3 + 2 min), support removal, airborne-particle abrasion with 50-µm glass beads (1.5 bar); AM-RC: cloth-cleaned in 95% ethanol, unpolymerized resin removed with cotton swab, air-dried; both postpolymerized	Maxillary first molar complete-coverage crown	Manufacturing accuracy (trueness and precision, RMS), intaglio surface adaptation (average gap distance, triple-scan protocol), survival after cyclic loading (1.2 million cycles, 5-year simulation)	AM-RC (Crowntec) had the highest overall manufacturing trueness and the highest trueness at internal occlusal, intaglio, and marginal surfaces; AM-HRC (VarseoSmile Crown Plus) had the lowest trueness at all surfaces. SM-PICN had the lowest precision. Intaglio adaptation was similar overall, but both printed materials showed better axial-wall adaptation than milled PICN. All crowns survived cyclic loading with no failures, cracks, or chipping.

							under nitrogen oxide gas (Otoflash G171) — 2 × 1500 flashes/surface (AM-HRC), 2 × 2000 flashes/surface (AM-RC)			
Abad-Coronel et al. (2023) [121]	In vitro comparative study	DPPa (permanent crown resin) vs 3DPPb (OnX); comparator: PMMA (milled)	SprintRay; Ivoclar Vivadent (PMMA comparator)	Hybrid definitive resin for 3D crown printing (3DPPa); nano-ceramic definitive hybrid (3DPPb); thermoplastic polymer (PMMA comparator)	No detailed chemical composition	3D-printing (SprintRay Pro-95); comparator: subtractive milling (CNC, MCX5)	Immersion in 90% alcohol (10 min, SprintRay Pro Wash/Dry), UV light curing for 9 min (SprintRay Pro Cure); all samples thermocycled (5000 cycles, 5–55°C)	Single-unit full-coverage crown	Fracture resistance, coefficient of variation	Milled PMMA showed the highest mean fracture resistance and lowest variability, followed by printed 3DPPa and then 3DPPb, with significant differences among all three materials. All three withstood average masticatory forces, supporting printed materials as a viable option for single-unit crowns.
Alharbi et al. (2021) [122]	In vitro comparative study	Irix Max (printed) vs Cerasmart (milled)	DWS (Italy); GC (Japan)	Definitive anohybrid composite resin; resin nanoceramic (comparator)	No detailed chemical composition	SLA (DFAB, DWS); comparator: subtractive milling (Ceramill motion 2)	Cleaned with 95% ethanol (1 min), UV post-cured (Dcure, DWS) for 5 min; printed at 180° build angle, 0.05 mm layer thickness	Incisal-wrap labial veneer (upper central incisor)	Stain susceptibility/color difference (ΔE00) after immersion in artificial saliva, tea and coffee (12 and 24 days), efficacy of stain removal (in-office bleaching vs surface polishing)	Printed veneers showed significantly higher stain susceptibility than milled veneers, with coffee and tea producing clinically unacceptable color changes; prolonged immersion increased discoloration. In-office bleaching removed stains more effectively than surface polishing in both groups. The printed material's color instability was judged unacceptable for permanent use, supporting only long-term temporary application.
Sabatini et al. (2024) [123]	In vitro comparative study	Crowntec (AM-D, definitive); comparators: FreeTEMP (AM-I, interim) and FreeTEMP + 3D-ADD CCA1 ceramic composite	Saremco AG (Crowntec); Detax (FreeTEMP); 3DResyns (concentrate)	Ceramic-filled resin (definitive); comparators: filler-free interim resin and ceramic-concentrate-modified interim resin	Ceramic-particle-filled resin for definitive use; interim resin with no fillers; interim resin mixed with 30 wt% ceramic composite concentrate	DLP (Asiga MAX UV)	AM-D: ethanol cloth cleaning; AM-I/AM-IR: ultrasonic cleaning in 98% isopropanol (3 + 3 min); all support-	Single molar crown (maxillary first molar)	Fabrication trueness (overall, external, intaglio, marginal RMS), internal fit (average gap, triple-scan protocol)	The ceramic-concentrate-modified interim resin (AM-IR) mostly showed the lowest trueness and highest internal gaps, whereas the definitive ceramic-filled resin (AM-D) performed comparably to or better than

		concentrate (AM-IR, modified interim)					removed and post-cured in Otoflash G171 (4000 flashes, 2000 × 2, nitrogen atmosphere)			the other groups. Differences among materials were small, and internal gaps for all groups remained below the reported clinical threshold (120 μm), suggesting clinically acceptable fit.
Li et al. (2025) [124]	In vitro study	Biomimetic gradient zirconia/resin composite (custom slurry: 3 mol% yttria-stabilized zirconia + HDDA/PETA resin)	Zirconia powder: Jinhan New Materials; monomers (HDDA, PETA, TPO): Aladdin Industrial Corporation; dispersant BYK-154: BYK	Zirconia/resin composite (ceramic-polymer)	DLP-printed 3Y-TZP porous zirconia scaffold infiltrated with dental resin (HDDA/PETA)	DLP (AUTOCERA-R)	Green body washed in anhydrous ethanol (ultrasonic), extended debinding, sintering per TGA (up to 1550°C); sintered scaffold piranha acid-etched (75% H_2SO_4 / 25% H_2O_2, 30 min), sonicated in ethanol, MDP-grafted, vacuum resin infiltration, light-cured (420–480 nm) 10 min, polished	Full crown	Manufacturing/dimensional accuracy of composite crown (deviation from design)	The biomimetic gradient zirconia/resin composite crown closely matched the design model (deviation 0.456 ± 0.124 mm) and showed good biocompatibility, supporting the feasibility of DLP-printed gradient crowns as a proof of concept.
Lim et al. (2025) [125]	In vitro study	TeraHarz TC-80DP (A2)	Graphy	Acrylate-based (methacrylate) resin	Methacrylate-based crown-and-bridge photopolymer resin	SLA / vat photopolymerization with heat-assisted (HVPP) modification (A1, Shindo — printer modified with custom heated resin tank; printed at 30, 40, 50°C vs untreated control)	Washed in 80% ethyl alcohol (10 min), dried under light blocking, post-cured 20 min at max 2,200 mW/cm² (300–550 nm; LC-3D Print Box, NextDent by 3D Systems)	Single crown (canine)	Three-dimensional accuracy (RMS deviation of printed crown vs CAD reference at different printing temperatures)	Printing temperature significantly affected crown accuracy: RMS deviation was highest at 50°C (median 113.20 μm), while 30°C and 40°C crowns were comparable to the untreated group. All groups remained within the 150 μm clinical threshold, but higher HVPP temperatures increased deviation, indicating a need to balance mechanical gains against dimensional accuracy.
Peskersoy & Acar (2025) [126]	Peskersoy & Acar (2025) [130]	VarseoSmile Crown Plus	BEGO	Ceramic-filled hybrid resin	Ceramic-filled permanent restorative resin (≈25 wt% filler)	DLP (Bego Varseo XS; 405 nm, 50 μm resolution)	Ultrasonic cleaning in 96% ethanol (480 s), light-microscopy	Inlay-retained 3-unit adhesive bridge (posterior,	Fracture resistance, color stability (ΔE_{00} after 5,000 thermal cycles), von Mises	The 3D-printed adhesive bridges showed the lowest fracture resistance of the three techniques

							inspection, post-cured in Bego Otoflash under nitrogen (1.0–1.2 bar), 1500 flashes at 10 Hz per side (both surfaces)	replacing mandibular second premolar)	stress and displacement (FEA)	(significantly lower than fiber-reinforced composite) but the best color stability, the only group within the clinically acceptable ΔE_{00} threshold. The authors concluded 3D-printed restorations are not yet suitable for the posterior region but may hold promise for lower-load anterior applications.
Farag et al. (2024) [127]	In vitro comparative study	Permanent Crown Resin (SLA) vs C&B resin (DLP)	Formlabs; NextDent	Hybrid composite resin (SLA, permanent); provisional C&B resin (DLP)	Hybrid composite permanent crown resin (Formlabs); methacrylate-based C&B resin (NextDent)	SLA (Form 2, Formlabs) and DLP (NextDent 5100); build angles 0°, 45°, 90°; layer thickness 50 µm	DLP: cleaned 5 min in 96% 2-propanol, cured 45 min (LC-3D Print Box); SLA: cleaned 1 min in 95% ethanol, post-cured 30 min UV (S2, DWS); all stored 10 days dry/light-proof	Single full-coverage crown (mandibular first molar)	Marginal discrepancy (influence of printing technology and build angle)	Both printer technology and build angle significantly affected marginal discrepancy. SLA produced significantly smaller marginal discrepancy (55.6 µm) than DLP (72 µm), and 0° build angle yielded the smallest discrepancy, followed by 45° then 90°. All values remained within the clinically acceptable 120 µm threshold.
Mangano et al. (2024) [128]	In vitro comparative study	Irix Max (printed) vs IPS e.max CAD (chairside milled) vs Natura Z Multilayer SMART (lab milled)	DWS Systems; Ivoclar Vivadent; DMAX Dental CAD/CAM Solutions	Ceramic-filled hybrid composite (printed); lithium disilicate and zirconia comparators	Hybrid composite filled with ceramic (~42 wt%), for definitive restorations; comparators: lithium disilicate, translucent monolithic zirconia	Tilted Stereolithography (TSLA; Dfab, DWS Systems); comparators: chairside 4-axis milling (inLab MC XL) and lab 5-axis milling (DWX-52D)	Washed in 95% ethyl alcohol (2–3 min, shaker), supports removed by hand, post-cured in dual-energy (UV + heat) Dcure device (~10 min cycle), staining/glazing and polishing per manufacturer	Single crown (premolar, #15)	Marginal trueness (ABS AVG, RMS, [90–10]/2 percentile), marginal adaptation/closure, occlusal and interproximal contact quality (prosthodontist rating), time efficiency, cost	The additive (TSLA hybrid composite) and lab-based milled zirconia crowns had significantly better marginal trueness than chairside-milled lithium disilicate; additive and lab-based did not differ significantly. Prosthodontist-rated marginal closure, interproximal, and occlusal contacts did not differ significantly across the three workflows. Time efficiency and cost trended in favor of the chairside additive workflow, supporting TSLA-printed hybrid composite as a valid option for definitive single crowns.

Baldi et al. (2025) [129]	In vitro comparative study	Irix Max (printed) vs VITA Enamic (chairside milled and industrially milled comparators)	DWS (Italy); VITA	Ceramic-filled hybrid composite (printed); polymer-filled hybrid ceramic (comparator)	Ceramic-filled hybrid composite (42 wt% ceramic); comparator: polymer-filled hybrid ceramic (86 wt% ceramic, 14 wt% UDMA/TEGDMA polymer)	Tilting stereolithography (TSLA; D-FAB, DWS); comparators: 4-axis chairside milling (Cerec MCXL) and 5-axis industrial milling (Micro 5x)	Washed for 1 min in a dedicated alcohol bath, light-cured for 3 min (DCURE, DWS)	Full-coverage adhesive overlay (upper premolar)	Volumetric interfacial adaptation, linear marginal adaptation, linear internal adaptation, external adaptation (micro-CT)	The chairside 3D printer showed the best volumetric adaptation and the chairside milling unit the worst. Internal adaptation was significantly worse for the chairside milling unit, while marginal (external) adaptation was comparable across all groups.
Shin et al. (2025) [130]	In vitro study	TC-80DP	Graphy Inc	Definitive resin	No detailed chemical composition	DLP (SprintRay Pro 95)	Rinsed in 95% isopropyl alcohol for 5 min (ultrasonic), outer and intaglio surfaces post-polymerized for 30 min (Cure-M 102H)	Single crown (maxillary first molar)	Marginal, axial, axio-occlusal, and occlusal gaps (replica technique); influence of cement space setting (35, 50, 70, 100 μm)	Cement space setting significantly affected marginal and internal fit. The 70-μm setting produced the smallest marginal, axio-occlusal, and occlusal gaps, while the 100-μm setting showed the largest axial gap. All marginal gaps remained within the clinically acceptable limit (<120 μm), and a 70-μm cement gap was recommended as optimal.
Kader et al. (2025) [131]	In vitro study	VarseoSmile TriniQ	BEGO (Germany)	Ceramic-filled hybrid resin	No detailed chemical composition	DLP (Asiga Ultra 50)	Washed in 99% isopropanol (5 min), post-cured twice for 20 min at 60°C (Form Cure)	Permanent maxillary molar endocrown	Marginal gap, internal gap, pulp chamber gap, overall gap (circumferential 3D analysis); influence of margin design (butt joint vs shoulder) and pulp chamber depth (2 vs 4 mm)	Preparation design significantly affected marginal, internal, and overall fit. Butt joint margins showed better marginal fit but poorer internal and overall fit, whereas a shoulder margin with 2-mm pulp chamber depth (Group 3) gave the best internal and overall adaptation. A 1-mm shoulder preparation with 2-mm pulp chamber depth was recommended for optimal fit.
Tartuk & Akın Tartuk (2026) [132]	In vitro comparative study	Crowntec (printed) vs Vita Suprinity PC (ZLS, milled) and Ceramill PEEK (milled)	Saremco Dental (Switzerland); VITA Zahnfabrik; Amann Girrbach	Definitive resin composite (printed); comparators: zirconia-reinforced lithium silicate glass-ceramic, PEEK	BisEMA (50–<75%), methyl benzoylformate (1–<5%); comparators: ZLS glass-ceramic (SiO_2 56–64%, Li_2O 15–21%, ZrO_2 8–12%), unfilled high-purity PEEK	SLA (Formlabs 3D printer); comparators: 5-axis milling	ost-cured under 405 nm UV for 30 min (Form Cure); intaglio airborne-particle abraded (50 μm Al_2O_3, 2 bar, 10 s), ultrasonically cleaned in	Molar endocrown (endodontically treated mandibular first molar)	Marginal gap (SEM), fracture resistance under axial load; effect of thermomechanical aging (5000 thermal cycles, 75,000 loading cycles)	The 3D-printed resin endocrowns showed the smallest marginal gaps of all groups, remaining within the clinically acceptable threshold (<120 μm) before and after aging. Thermomechanical aging significantly increased marginal gaps and

							ethanol (3 min), air-dried			significantly reduced fracture resistance of the printed resin (−20.9%), which had the lowest fracture resistance among the three materials. The authors cautioned that printed resins are susceptible to mechanical degradation after aging.
Tartuk et al. (2026) [133]	In vitro comparative study	Crowntec (printed) vs Vita Suprinity PC (ZLS, milled)	Saremco Dental (Switzerland); VITA Zahnfabrik	Definitive resin composite (printed); comparator: zirconia-reinforced lithium silicate glass-ceramic	BisEMA (50–<75%), methyl benzoylformate (1–<5%)SLA (Formlabs 3D printer); comparator: 5-axis milling (Ceramill Motion 2); comparator: ZLS glass-ceramic (SiO_2 56–64%, Li_2O 15–21%, ZrO_2 8–12%)	SLA (Formlabs 3D printer); comparator: 5-axis milling (Ceramill Motion 2)	Washed in 95% isopropyl alcohol for 3 min (Form Wash), post-cured under 405 nm LED at 60°C for 20 min (Form Cure); after support removal, an additional curing cycle under the same parameters; intaglio airborne-particle abraded (50 μm Al_2O_3, 0.15 MPa)	Occlusal overlay (mandibular first molar)	Marginal gap (SEM), fracture resistance; influence of preparation design (O vs OS) and thermomechanical aging (75,000 loading cycles, 5500 thermal cycles)	Preparation design significantly affected both outcomes, whereas fabrication technique/material type did not significantly affect marginal gap. The printed resin overlays showed marginal adaptation comparable to milled ZLS, with occlusal-reduction-only designs giving smaller marginal gaps than round-shoulder designs; all groups remained within the clinically acceptable limit (<120 μm). Printed resin had significantly lower fracture resistance than ZLS but exceeded physiological occlusal forces, supporting its potential as a conservative overlay alternative.
Kahraman & Özden (2026) [134]	In vitro comparative study	VarseoSmile Crown Plus; P-Crown Version 3; Saremco Print Crowntec	BEGO; Senertek; Saremco Dental	Definitive resin composites (high-viscosity hybrid resins and glass-filler-reinforced composite resin)	VarseoSmile Crown Plus — hybrid resin, 30–50 wt% dental glass/silica fillers; P-Crown Version 3 — 65 wt% nano-ceramic filler; Saremco Print Crowntec — glass-filler-reinforced composite, up to 50 wt% silanized dental glass and pyrogenic silica (0.7 μm)	LCD (Phrozen Sonic Mini 8K)	Cleaned in 99% isopropyl alcohol; post-polymerized under nitrogen atmosphere (0.15 MPa) at 2000, 4000, or 6000 flashes (Otoflash G171)	One-piece endodontic crown (mandibular first molar)	Trueness (RMS, mean positive and negative deviation, total match percentage); influence of resin type and post-polymerization duration	Post-polymerization duration (2000/4000/6000 flashes) had no significant effect on trueness for any resin. Resin type significantly affected trueness: the glass-filler-reinforced composite (Crowntec) showed the lowest RMS and highest total match percentage, followed by VarseoSmile Crown Plus and then P-Crown Version 3. All deviation values remained below 100 μm, and appropriate resin selection

										was more influential than post-curing duration.
Güven et al. (2024) [135]	In vitro comparative study	Tera Harz TC-80DP (definitive printed); FREEPRINT temp (interim printed); comparator: Tetric CAD (milled)	Graphy; Detax; Ivoclar Vivadent	Definitive acrylate resin (printed); comparators: interim methacrylate resin, milled composite resin	R — urethane acrylate oligomer, bisphenol A ethoxylate dimethacrylate, 2-HEMA, diphenyl (2,4,6-trimethylbenzoyl) phosphine oxide; FT — isopropylidenediphenol PEG-2 dimethacrylate (45–<60 wt%), 2-hydroxyethyl methacrylate, diphenyl (2,4,6-trimethylbenzoyl) phosphinoxide, hydroxypropyl methacrylate; comparator TC — Bis-GMA, ethoxylated Bis-GMA, TEGDMA, UDMA, barium glass (<1 µm), silicon dioxide (<20 nm)	DLP (Asiga MAX UV, 50 µm layer thickness); comparator: subtractive milling (PrograMill PM7)	FT — ultrasonic cleaning in 98% isopropyl alcohol (3 + 3 min), air-dried; GR — ultrasonic cleaning in 96% ethanol (40 s), unpolymerized resin removed with ethanol-soaked cotton swab, air-dried; both post-polymerized under nitrogen oxide gas (Otoflash G171, 2000 × 2 exposures); no polishing	Mandibular first molar onlay	Dimensional stability (external surface deviation, mesiodistal width deviation), occlusal surface wear, fracture resistance, Weibull analysis, failure behavior after thermomechanical aging	The additively manufactured definitive resin (GR) showed the highest external surface deviation and occlusal surface wear, while the interim resin (FT) had the greatest mesiodistal width deviation. Subtractively manufactured composite (TC) exhibited the lowest wear, highest fracture resistance, and highest reliability. Both printed resins had comparable fracture resistance, and all materials exceeded reported masticatory forces, supporting their use for onlays at 1.5-mm thickness, though printed onlays may be more prone to occlusal and interproximal contact issues.
Aziz et al. (2025) [136]	In vitro comparative study	VarseoSmile Crown plus (3D-VS, printed) vs 3D Pro Zir (M-Zr, milled) vs IPS e.max Press (P-LD, pressed)	Bego; Aidite Technology; Ivoclar Vivadent	Ceramic-reinforced composite resin (printed); comparators: zirconia, pressed lithium disilicate	VarseoSmile Crown plus — inorganic fillers (0.7 µm particle size) 30–50 wt%, 4,4′-isopropylidiphenol ethoxylated, 2-methylprop-2enoic acid, silanized dental glass, methyl benzoylformate, diphenyl (2,4,6-trimethylbenzoyl) phosphine oxide; comparators: 3D Pro Zir — ZrO_2 90–95%, Y_2O_3 4–10%, Al_2O_3 ≤0.5%; IPS e.max Press — SiO_2, Li_2O, K_2O, P_2O_5, ZrO_2, ZnO, coloring oxides	DLP (Varseo XS, 30° build angle); comparators: 5-axis milling (AMD 500DC), heat pressing (Meta-press)	Support removal with side cutter, ultrasonic cleaning in 96% ethanol (45 s), polymerized in light-polymerizing unit (Sibari SR620); steam-jet cleaned, support remnants removed under magnification loupes, surfaces smoothed	Maxillary incisor laminate veneer (shoulder margin and feather-edge designs)	Marginal adaptation (cervical, mesial, distal, incisal), internal adaptation (cervical, middle, incisal thirds), trueness; influence of preparation design (shoulder margin vs feather-edge)	Milled zirconia (M-Zr) showed the best marginal and internal adaptation and trueness among all groups, while the printed hybrid resin (3D-VS) generally showed the highest discrepancies. The shoulder margin design consistently produced better fit than the feather-edge design across all three materials. All marginal and internal adaptation values for the three materials remained within the clinically acceptable range.
Sasany & Al-Johani (2026) [137]	In vitro comparative study	FREEPRINT Crown (unmodified control); PMMA + 1 wt% SiO_2 (S); PMMA + 2 wt% TiO_2 (T);	Detax GmbH & Co. KG; SiO_2 (AEROSIL 200) and TiO_2	Polymethyl methacrylate (PMMA) crown resin, unmodified	Meth)acrylic resin with phosphine oxides and silanized dental glass, 20–40 wt% inorganic fillers (mean filler size ~1.5 µm); reinforced with amorphous SiO_2 nanoparticles (~12	DLP (Asiga MAX UV, 50 µm layer thickness, 90° build orientation)	Support structures removed with cut-off wheel; external surfaces	Mandibular first molar complete crown	Fabrication trueness (marginal, intaglio, total RMS), color stability (ΔE_{00}), surface roughness (Ra), wear (occlusal RMS after	Co-reinforcement with SiO_2 and TiO_2 (ST) produced the highest fabrication trueness, lowest color change, smoothest surface, lowest

		PMMA + 1 wt% SiO_2 + 2 wt% TiO_2 (ST)	(AEROXIDE TiO_2 P25): Evonik Industries	and nanoparticle-reinforced	nm, 1 wt%) and/or mixed anatase/rutile TiO_2 nanoparticles (~21 nm, 2 wt%)		finished with progressively finer silicon carbide papers (500–2000 grit) under water; cleaned with 98% isopropyl alcohol on lint-free cloth; UV polymerization in light-polymerizing chamber (Otoflash G171); intaglio airborne-particle abraded (50 μm Al_2O_3, 0.2 MPa)		thermomechanical aging, JET defect-probability heatmaps), fracture resistance	wear, and highest fracture resistance among all groups. TiO_2 alone (T) improved fracture resistance and wear but increased color change, while SiO_2 alone (S) mainly improved surface characteristics without significantly enhancing fracture resistance. The synergistic combination of both nanoparticles enhanced the overall optical, surface, and mechanical performance of AM PMMA crowns.
Altinci et al. (2025) [138]	In vitro comparative study	Crowntec (AM-CT); FREEPRINT Crown (AM-FP); Tera Harz TC-80DP (AM-GR); comparator: Tetric CAD (SM-TC, milled)	Saremco Dental AG; Detax; Graphy; Ivoclar AG	Definitive resin composites (printed); comparator: milled glass-reinforced composite resin	M-CT — hybrid resin with ceramic fillers (esterification products of 4,4′-isopropylphenol ethoxylated and 2-methylprop-2enoic acid, silanized dental glass, pyrogenic silica), 30–50 wt% fillers; AM-FP — hybrid resin with ceramic fillers (alkoxylated phenol derivative, methacrylate-terminated bismethacrylate, 1,6-hexanediol dimethacrylate), 20–40 wt% fillers; AM-GR — urethane acrylate-based resin without fillers (urethane acrylate oligomer, bisphenol A ethoxylate dimethacrylate, 2-HEMA); comparator SM-TC — barium glass filler, silicon dioxide, dimethacrylates, 71.1 wt% fillers	DLP (Asiga MAX UV, 50 µm layer thickness); comparator: 5-axis milling (PrograMill7)	AM-CT — cleaned with 96% ethanol-soaked cloths; AM-FP — isopropyl alcohol (≥98%, 3 + 2 min); AM-GR — 96% ethanol ultrasonic (45 s) then ethanol-soaked cloth; all air-dried and post-polymerized under nitrogen oxide gas (Otoflash G171, 4000 exposures); supports removed and smoothed with finishing burs, external surfaces left unpolished	Mandibular first molar endocrown (butt joint or shoulder margin; 2 mm or 4 mm pulp chamber depth)	Fabrication trueness (external, intaglio, marginal RMS), internal fit (average gap, triple scan protocol); influence of material type, margin configuration, and pulp chamber depth	Fabrication trueness and fit were significantly influenced by material type, margin configuration, and pulp chamber depth, with no single subgroup showing consistently highest trueness. AM-GR endocrowns had the lowest external but higher intaglio/marginal trueness and better fit, while SM-TC had the highest external but lower intaglio/marginal trueness and poorer fit. Only SM-TC with 4 mm pulp chamber depth exceeded the 120 µm fit threshold; most endocrowns showed clinically acceptable internal gaps.
Çakmak et al. (2024) [139]	In vitro study	Tera Harz TC-80DP	Graphy	Definitive resin (urethane acrylate-based)	Urethane acrylate-based definitive resin	DLP (Asiga MAX UV, 50 µm layer thickness,	Left to drip excess resin (10 min),	Maxillary central incisor laminate	Fabrication trueness (overall, external, intaglio, marginal RMS), margin	0.7 mm-thick veneers had significantly higher fabrication trueness (lower

						perpendicular build orientation)	ultrasonically cleaned in 96% ethanol (45 s) then cleaned with 96% ethanol-soaked cloth, air-dried, polymerized under nitrogen oxide gas (Otoflash G171, 4000 exposures); supports removed under magnification loupes, no additional adjustments	veneer (0.5 mm and 0.7 mm thickness)	quality (labial, incisal, mesial, distal); influence of restoration thickness and margin location	deviations) than 0.5 mm-thick veneers across all surfaces. Restoration thickness did not affect margin quality, but labial margins had the lowest quality among all locations. Tested resin veneers may require less adjustment at 0.7 mm thickness, though marginal integrity issues may occur at the labial surface.
Kakinuma et al. (2022) [140]	In vitro comparative study	TNG-GPM101 (3D-printed) vs CERASMART Prime (milled)	GC	Hybrid resin-composite	Hybrid resin-composite liquid (printed); comparator: hybrid resin-composite block (milled)	DLP (Asiga MAX UV, 0.05 mm layer thickness, occlusal surface parallel to build platform, 385 nm UV laser); comparator: wet milling (Aadva LW-I), with un-offset (0.0 mm) and offset (0.5 mm) drill settings	Ultrasonic cleaning in 96% isopropanol (2 min, repeated twice), build supports removed, inner surface sandblasted with 50 µm alumina (0.15 MPa); polymerized with UV-light curing unit (Otoflash G171, 3000 flashes)	Mandibular first molar full crown	Dimensional accuracy/trueness (RMS at occlusal, buccal, lingual, cusp), marginal discrepancy, internal gap (cement space at cusp tip); influence of abutment shape and milling drill-offset	3D-printed crowns showed significantly higher dimensional accuracy (RMS <20 µm median, <50 µm all areas) with fewer marginal discrepancies than milled crowns regardless of abutment form. Milled crowns showed significant deviations, especially at cusps, and offset correction created internal grooves with negative deviations that were most evident in sharp abutment models. DLP-based 3D printing accommodated a wider range of abutment shapes, including sharp occluso-axial line angles unsuitable for milling.
Orgev et al. (2026) [141]	In vitro comparative study	Pro Resins Crown X (AM-S); Flexcera Smile Ultra+ (AM-F); comparators: Shofu HC (SM-S); Brilliant Crios (SM-C, milled)	Straumann USA LLC; Desktop Health; SHOFU Dental Corp; Coltène AG	Definitive resin composites (glass-filled resin-based composite and hybrid nanoceramic resin); comparators:	Pro Resins Crown X — glass-filled resin-based composite (acrylic resin, UDMA, diacrylate, TPO); Flexcera Smile Ultra+ — hybrid nanoceramic ceramic-filled resin (10–20% methacrylated monomer, 25–40% methacrylic oligomer, 25–40% methacrylated oligomer, 1–10%	DLP (Straumann P30 / CARES P30); comparator: subtractive milling (Ivoclar PM7, wet milling)	AM crowns post-processed with P-wash unit for 20 min in 99% isopropyl alcohol and light-polymerized in	Maxillary first molar complete-coverage crown	Fracture resistance after fatigue cyclic loading (1.2 million cycles, 49 N), load-to-fracture, failure mode	All crowns survived cyclic loading. The subtractively manufactured Shofu HC (SM-S) showed significantly lower fracture resistance (2184 ± 660.2 N) than the other three groups, which exceeded 2500 N with no

				hybrid ceramic and glass-reinforced composite (milled)	methacrylated monomer, 0.1–1% TPO); comparators: Shofu HC — hybrid ceramic (UDMA, TEGDMA, silica, zirconium silicate); Brilliant Crios — glass-reinforced composite (dental glass, cross-linked methacrylates, amorphous silica)		P-cure unit; support removal under magnification, no additional polishing; steam-cleaned. For cementation: airborne-particle abrasion of crown with 50-µm Al_2O_3; adhesive cementation with Monobond Plus primer and Variolink Esthetic DC			significant differences among them. Both printed materials demonstrated fracture resistance comparable to the milled comparators and above clinically acceptable thresholds, supporting their potential for definitive tooth-supported crowns.
Çakmak et al. (2024) [142]	In vitro comparative study	VarseoSmile Crown Plus (AM-B); Crowntec (AM-S); comparator: Vita Enamic (SM, milled)	Bego; Saremco; comparator: Vita Zahnfabrik	Additively manufactured hybrid resin composite and resin composite; comparator: polymer-infiltrated ceramic network (milled)	VarseoSmile Crown Plus — esterification products of 4,4′-isopropylidiphenol ethoxylated and 2-methylprop-2-enoic acid, silanized dental glass, methyl benzoylformate, diphenyl (2,4,6-trimethylbenzoyl) phosphine oxide, inorganic filler content 30–50 wt%; Crowntec — esterification products of 4,4′-isopropylidiphenol ethoxylated and 2-methylprop-2-enoic acid, silanized dental glass, pyrogenic silica, initiators, inorganic filler content 30–50 wt%; comparator: Vita Enamic — 86 wt% fine-structure feldspar ceramic network + 14 wt% methacrylate polymer (UDMA/TEGDMA)	DLP (ASIGA MAX UV); comparator: subtractive milling (PrograMill PM7)	AM-B — ultrasonic cleaning in 95% ethanol (reusable then fresh bath), air-dried, support removal, sandblasting with 50 µm glass beads (1.5 bar), post-polymerized under nitrogen (Otoflash G171, 2 × 1500 flashes); AM-S — 95% ethanol cloth cleaning, air-dried, post-polymerized under nitrogen (Otoflash G171, 2 × 2000 flashes), then sandblasted with 50 µm glass beads and support removal; no	Mandibular first molar crown (2-mm occlusal thickness)	Fracture resistance; influence of restorative material, resin cement (RelyX Universal vs Panavia V5), and cyclic loading (1.7 Hz, 1.2 million cycles, 49 N)	Material, resin cement, and cyclic loading each significantly affected fracture resistance, with no significant interactions. The milled polymer-infiltrated ceramic network (SM) had the highest fracture resistance and the printed VarseoSmile Crown Plus (AM-B) the lowest, while RelyX Universal produced higher values than Panavia V5 and cyclic loading reduced fracture resistance. All materials exceeded reported molar masticatory forces, supporting their reliability for single molar crowns with 2-mm occlusal thickness in the short-to-mid term.

							glazing or polishing. For cementation: intaglio sandblasted with 50 µm Al_2O_3, cemented with RelyX Universal or Panavia V5			
Abuabboud et al. (2025) [143]	Pilot in vitro study	SprintRay Crown	SprintRay Inc.	Resin-based permanent restorative material	No detailed chemical composition	Masked stereolithography (MSLA; Prusa SL1)	Post-processed, sandblasted, and glazed; for cementation: intaglio polished, ultrasonically cleaned, sandblasted with 50 µm alumina, cleaned with anhydrous alcohol, Scotchbond Universal Plus Adhesive, cemented with RelyX Universal Resin Cement	Molar endocrown	Fracture resistance; influence of cement layer thickness, intracoronal depth, and dentine wall thickness (CBCT-based measurements), failure mode	Cement layer thickness showed a strong positive correlation with fracture resistance (r = 0.577) and was the only variable reaching statistical significance (ANOVA F = 7.847, p = 0.019; confirmed by Mann–Whitney U, p = 0.015), with the thicker-cement group (≥0.3 mm) averaging higher fracture force than the thinner group. Intracoronal depth and dentine wall thickness showed weaker, nonsignificant correlations. Fractures frequently initiated at the cement–dentine interface, indicating cement thickness is a key biomechanical determinant of 3D-printed endocrown fracture resistance while preparation depth and dentine geometry have a less direct impact.
Arslan Acicbe et al. (2025) [144]	In vitro comparative study+C56D57	VarseoSmile Triniq (printed) vs breCAM.HIPC (milled)	Bego; bredent	Definitive restorative resin (printed); high-impact polymer composite (milled)	Esterification products of 4,4′-isopropylidenediphenol, ethoxylated, and 2-methyl-prop-2-enoic acid (55–80 wt%); benzeneacetic acid, .alpha.-oxo-, methyl ester (<5 wt%); diphenyl(2,4,6-trimethylbenzoyl)phosphine oxide (<2.5 wt%); titanium dioxide (<2.5 wt%). Comparator: ultracompact polymethylmethacrylate with ceramic microfillers	Low force display (LFD; Form 4B, Formlabs), digital light processing (DLP; Max UV, Asiga), and liquid crystal display (LCD; SOL, Ackuretta); comparator: five-axis subtractive milling (Ceramill Motion 2, Amann Girrbach)	Ultrasonic cleaning in 96% ethanol for 5 min (3 min prewash + 2 min postwash), air-drying, support removal, post-polymerization in Form Cure (Formlabs) at 60 °C for 20 min,	hree-unit posterior FDP (maxillary first premolar and first molar abutments)	Fabrication trueness (external, intaglio, and marginal surface deviations; RMS), fit (average gap, triple-scan protocol), precision	AM-LFD showed the highest external surface trueness, while SM had the highest intaglio trueness and the lowest marginal trueness; AM-LCD produced the lowest trueness overall. None of the printed FDPs achieved gaps comparable to the cement space, and all exceeded the clinical threshold regardless of

							repeated twice; 45-degree build orientation, 50 µm layer thickness			printer, whereas SM FDPs had the best, clinically acceptable fit.
Revilla-León et al. (2025) [145]	In vitro study	Permanent Crown (A2 V1)	Formlabs	Definitive resin-ceramic	No detailed chemical composition	Stereolithography (SLA; Form 3B+, Formlabs)	Separation from build platform; immersion in 95% pure ethyl alcohol for 3 min in a rinsing unit (Form Rinse, Formlabs); toothbrush cleaning of intaglio surface; air-drying followed by 30 min drying on paper towel at 24 °C; first post-polymerization cycle 20 min at 60 °C (Form Cure, Formlabs); intaglio airborne-particle abrasion with glass beads (Perlablast, Bego) at 0.15 MPa; second post-polymerization cycle 20 min at 60 °C; print orientations tested 0°, 45°, 70°, and 90°; 50 µm layer thickness	Anatomic contour molar crown	Intaglio surface fabricating trueness and precision (RMS error), influence of print orientation	Print orientation significantly affected the intaglio surface trueness and precision of the definitive resin-ceramic crowns. The 0-degree orientation produced the best trueness (37 µm) and highest precision, whereas the 90-degree orientation gave the worst trueness (113 µm), supporting a 0-degree orientation for this SLA printer and material.
Cao et al. (2025) [146]	In vitro study	Crowntec vs Varseosmile Crownplus vs NextDent C&B MFH (printed); Tetric CAD (milled control)	Saremco; Bego; NextDent Systems; Ivoclar Vivadent	3D Glass filler-reinforced resin composite (Crowntec); ceramic-filled hybrid resin	Crowntec — BisEMA (50–<70%), trimethylbenzoyldiphenylphosphine oxide (0.1–<1%). Varseosmile Crownplus — esterification products of 4,4′-isopropylidenediphenol, ethoxylated, and 2-methylprop-2-	Digital light processing (DLP; Asiga Max UV, Asiga); comparator: subtractive milling	Printed at 50 µm layer thickness, 45° print orientation. Hybrid resin composites	Mandibular left first molar full-coverage single crown	Dimensional accuracy (RMS trueness, positive and negative average deviations) of intaglio, marginal, and interproximal contact surfaces; influence	Post-polymerization method affected the dimensional accuracy of the AM crowns, with post-polymerizing in glycerine alongside the manufacturer's

				(Varseosmile Crownplus); long-term temporary resin (NextDent C&B MFH); comparator: resin composite (Tetric CAD)	enoic acid (50.00–<75.00 wt%), diphenyl(2,4,6-trimethylbenzoyl)phosphine oxide (<2.5 wt%). NextDent C&B MFH — methacrylic oligomer (>60 wt%), glycol methacrylate (15–25 wt%), phosphine oxide (<2.5 wt%). Comparator: resin composite (composition not published)	(PrograMill PM7, Ivoclar)	(Varseosmile Crownplus, NextDent C&B MFH) rinsed in 99.5% ethanol for 3 min + 2 min clean ethanol, air-dried; Crowntec cleaned with alcohol-soaked brush and kept in dark. Supports removed with scalpel. Post-polymerized in one of four units (Cara Print LEDcure/Kulzer at 60 °C; LC-3DPrint Box/NextDent at 60 °C; Otoflash G171/NK-Optik at room temperature, 2000×2 flashes; SES Curing Light/INNO DENTAL at room temperature) for 30 min, under three conditions (UV only, UV + glycerine, or UV + nitrogen); stored in dark ≥24 h		of post-polymerization unit, inert medium (glycerine, nitrogen), and temperature; SEM surface analysis	recommended unit generally improving accuracy, while post-polymerization temperature (20 °C vs 60 °C) had no significant effect. The milled control showed the highest accuracy for intaglio and marginal surfaces, though interproximal contacts did not differ significantly from AM crowns. Crowntec consistently showed the highest RMS values, sometimes exceeding the clinically acceptable 100–120 µm range.
Demirel et al. (2025) [147]	In vitro comparative study	Irix Max (AM-IX); Irix Plus (AM-IP); VarseoSmile Crown Plus (AM-VS); Tera Harz TC-80DP (AM-GR); comparator:	DWS Systems; Bego; Graphy; comparator: Ivoclar	Definitive resin composites (hybrid composite and urethane acrylate resins)	Irix Max — hybrid composite resin with 42 wt% ceramic fillers (composition not disclosed); Irix Plus — hybrid composite resin with 36 wt% ceramic fillers (composition not disclosed); VarseoSmile Crown Plus — hybrid composite resin with 30–50	Tilting stereolithography (TSLA; DFAB, DWS Systems) for Irix Max and Irix Plus; DLP (Asiga MAX UV) for VarseoSmile Crown	AM-IX/AM-IP: cleaned in 96% ethanol (2 min, shaker), support removal, nine-minute post-cure (DCure, dual-	Ultrathin maxillary central incisor laminate veneer (0.3 mm and 0.5 mm thickness)	Fabrication trueness (overall, external, intaglio, marginal RMS), fit (average gap, triple-scan protocol), margin quality; influence of manufacturing trinomial (technology, printer,	The manufacturing trinomial and restoration thickness affected fabrication trueness, fit, and margin quality. Veneers printed with the tilting stereolithography printer generally showed

		Tetric CAD (SM-TC, milled)			wt% ceramic fillers (esterification products of 4,4′-isopropylphenol ethoxylated and 2-methylprop-2-enoic acid, silanized dental glass, methyl benzoylformate, diphenyl (2,4,6-trimethylbenzoyl) phosphine oxide); Tera Harz TC-80DP — urethane acrylate-based resin with no fillers; comparator Tetric CAD — Bis-GMA, UDMA, Bis-EMA, TEGDMA, barium glass (<1 µm), silicon dioxide (<20 nm), 71 wt% inorganic fillers	Plus and Tera Harz TC-80DP; comparator: 5-axis milling (PrograMill PM7); all at 50 µm layer thickness	energy UV+heat). AM-VS: ultrasonic 96% ethanol (3+2 min), support removal, airborne-particle abrasion (50 µm glass beads, 0.15 MPa), post-cured under nitrogen (Otoflash G171, 2×1500 flashes). AM-GR: ultrasonic 95% ethanol (45 s) then ethanol-soaked microbrushes, post-cured under nitrogen (Otoflash G171, 2×2000 flashes), support removal. No polishing		material) and restoration thickness	higher trueness than DLP-printed and milled veneers, with Irix Plus mostly yielding the highest trueness and lowest gaps. Thinner (0.3 mm) veneers had improved fit. All tested veneers achieved clinically acceptable fit, and most showed slightly rough margins with small defects.
Peng et al. (2026) [148]	In vitro study	FREEPRINT crown (A2)	DETAX	Definitive resin (permanent crown resin)	No detailed chemical composition	DLP (DMG 3Demax, 385 nm, build angle 0°)	Washed in 95% isopropyl alcohol (3Dewash) for 180, 210, or 240 s; air-dried; post-polymerized 30 min (3Decure); support removal (crowns cleaned for 210 s)	Single crown (maxillary right first premolar; thicknesses 1.25, 1.50, 1.75, 2.0 mm)	Fracture load (influence of crown thickness and thermal aging), marginal and internal fit (micro-CT; influence of thermal aging), failure mode	Fracture load increased with crown thickness but decreased significantly after thermal aging (10,000 cycles), dropping from 1240.3 N to 955.3 N for 2.0-mm crowns. Marginal and internal gaps remained within the clinically acceptable range and decreased after aging (attributed to moisture-induced expansion). A 2-mm thickness was recommended for durability under maximum clenching forces, and thermal aging had a more pronounced negative effect on mechanical performance than cleaning time.

Nagai et al. (2025) [149]	In vitro comparative study	VarseoSmile Crown Plus (printed definitive, DLP and SLA); comparators: Cerasmart (milled definitive nano-ceramic), plus interim materials DENTCA Crown & Bridge resin and Vericore Gradient Temp PMMA	BEGO; comparators: GC America; DENTCA; Whip Mix	Ceramic-filled hybrid resin (printed definitive); comparator: milled nano-ceramic hybrid	VarseoSmile Crown Plus — esterification products of 4,4′-isopropylidiphenol ethoxylated and 2-methylprop-2-enoic acid, silanized dental glass, methyl benzoylformate, diphenyl (2,4,6-trimethyl benzoyl) phosphine oxide; 30–50 wt% inorganic fillers (0.7 µm); comparator Cerasmart — Bis-MEPP, UDMA, DMA, 71% silica and barium glass nanoparticles	DLP (Varseo XS) and SLA (Form 3) for the definitive printed material; comparator: 5-axis milling (DWX-51D)	Manufacturer-recommended nesting, printing, and post-processing performed by a certified dental laboratory; before cementation crowns sandblasted with 50 µm alumina (0.2 MPa); nano-filled protective coating (Optiglaze) applied to all crowns and light-polymerized 3 min	Full-coverage single definitive crown (maxillary, 12 mm × 10 mm, 1.5 mm thickness)	Color stability (ΔE_{00}, CIEDE2000) after immersion in artificial saliva and wine at 1, 3, and 6 months; comparison against acceptability (AT) and perceptibility (PT) thresholds	Wine caused significant discoloration exceeding acceptability and perceptibility thresholds in the printed hybrid ceramic-filled definitive crowns (both DLP and SLA) at all time intervals, whereas milled nano-ceramic definitive crowns showed the greatest color stability. The type of 3D printer (DLP vs SLA) did not significantly affect color stability of the same material. In artificial saliva, 80–100% of samples remained within acceptable and perceptible thresholds; the authors concluded printed hybrid ceramic-filled definitive crowns may be unsuitable for patients with heavy wine consumption.

Supplementary Table S3. Study characteristics and extracted data for studies directly comparing additively manufactured zirconia and resin materials within the same investigation (n = 2).

Author (Year)	Study Design	Material	Manufacturer	Material Category	Material Composition	Printing Technology	Post-Curing Protocol	Restoration Type	Outcomes Evaluated	Main Findings
Sasany et al. (2025) [150]	In vitro comparative study	CROWNTEC (printed) vs LithaCon 3Y 210 (printed) vs CEREC Tessera (milled)	Saremco Dental (Switzerland); Lithoz (Austria); Dentsply Sirona (Germany)	Ceramic-infused definitive resin composite; zirconia ceramic; advanced	Glass-filler-reinforced resin composite; 3 mol% yttria-stabilized zirconia (3Y-TZP); advanced lithium disilicate glass-ceramic (comparator)	DLP (Asiga MAX UV) for composite; LCM (CeraFab 7500) for zirconia; comparator: subtractive milling (CEREC Primemill)	Composite: ethanol wiping, dried 10 min, post-cured in Otoflash G171 (2000 flashes/surface under nitrogen). Zirconia: LithaSol cleaning, debinding to 600°C, sintering at 1450°C for 2 h.	Molar endocrown	Marginal and internal adaptation, fabrication trueness (RMS), retention (pull-out force), failure mode	Printed zirconia and milled advanced lithium disilicate showed comparable marginal and internal adaptation and trueness, both significantly better than

				lithium disilicate			Three-step material-specific polishing for all groups.			printed composite. Printed zirconia demonstrated the highest retention, significantly greater than lithium disilicate and composite. AM zirconia appears a viable alternative to SM lithium disilicate for endocrowns.
Sasany et al. (2025) [151]	In vitro comparative study	Crowntec (AM ceramic-filled resin); LithaCon 3Y 210 (AM zirconia); comparators: Lava Ultimate (SM nano-ceramic resin), VITA YZ HT (SM zirconia)	Saremco Dental (labeled Dentona in Table 1); Lithoz; 3M ESPE; VITA Zahnfabrik	Ceramic-filled resin and zirconia ceramic	Crowntec — methacrylate-based resin with BisEMA (50–<70%), trimethylbenzoyldiphenylphosphine oxide (0.1–<1%), ceramic nano/micro-fillers; LithaCon 3Y 210 — 3 mol% yttria-stabilized zirconia in light-curing resin matrix (~60 vol% polymer); comparators: Lava Ultimate — 80 wt% nano-ceramic filler + 20 wt% resin; VITA YZ HT — 3 mol% yttria-stabilized zirconia (3Y-TZP)	DLP (Asiga MAX UV) for ceramic-filled resin; LCM (Lithoz CeraFab 7500) for zirconia; comparator: 5-axis milling (Ceramill Motion 2 5x)	Ceramic-filled resin: ethanol-soaked cloth cleaning, air-dried 10 min, post-cured under nitrogen (Otoflash G171, 2000 flashes/surface). Zirconia: support removal, LithaSol spray cleaning, sintering (Programat CS4, peak 840°C, 10 min hold). Occlusal surfaces finished with fine diamond bur and three-step polishing; intaglio left untreated	0.5-mm occlusal veneer (maxillary first molar)	Fracture resistance, wear resistance (volumetric and vertical wear), bond strength; influence of polydopamine (PDA) surface treatment, after thermomechanical aging (5 million cycles)	Subtractively manufactured zirconia showed the best overall mechanical performance, while untreated printed ceramic-filled resin performed the worst. Polydopamine treatment significantly improved fracture resistance and bond strength in both printed materials but had minimal effect on milled ceramics; it reduced wear in the printed groups but unexpectedly increased wear in milled nano-ceramic resin. All materials at 0.5-mm thickness withstood clinically relevant occlusal forces.
